# Alternative Splicing of Barley Clock Genes in Response to Low Temperature

**DOI:** 10.1371/journal.pone.0168028

**Published:** 2016-12-13

**Authors:** Cristiane P. G. Calixto, Craig G. Simpson, Robbie Waugh, John W. S. Brown

**Affiliations:** 1 Division of Plant Sciences, School of Life Sciences, University of Dundee at the James Hutton Institute, Invergowrie, Dundee, Scotland; 2 Cell and Molecular Sciences, The James Hutton Institute, Invergowrie, Dundee, Scotland; International Centre for Genetic Engineering and Biotechnology, ITALY

## Abstract

Alternative splicing (AS) is a regulated mechanism that generates multiple transcripts from individual genes. It is widespread in eukaryotic genomes and provides an effective way to control gene expression. At low temperatures, AS regulates Arabidopsis clock genes through dynamic changes in the levels of productive mRNAs. We examined AS in barley clock genes to assess whether temperature-dependent AS responses also occur in a monocotyledonous crop species. We identify changes in AS of various barley core clock genes including the barley orthologues of Arabidopsis *AtLHY* and *AtPRR7* which showed the most pronounced AS changes in response to low temperature. The AS events modulate the levels of functional and translatable mRNAs, and potentially protein levels, upon transition to cold. There is some conservation of AS events and/or splicing behaviour of clock genes between Arabidopsis and barley. In addition, novel temperature-dependent AS of the core clock gene *HvPPD-H1* (a major determinant of photoperiod response and *AtPRR7* orthologue) is conserved in monocots. *HvPPD-H1* showed a rapid, temperature-sensitive isoform switch which resulted in changes in abundance of AS variants encoding different protein isoforms. This novel layer of low temperature control of clock gene expression, observed in two very different species, will help our understanding of plant adaptation to different environments and ultimately offer a new range of targets for plant improvement.

## Introduction

Alternative splicing (AS) of pre-mRNA transcripts is where the differential use of splice sites produces different mRNA transcripts from the same gene [1,2]. It is a widespread phenomenon in higher eukaryotes and generates transcriptome and proteome diversity [3]. The biological roles of AS are diverse, contributing to eukaryote complexity and shaping their evolution [4–8]. In plants, AS occurs frequently in more than 60% of intron-containing genes in Arabidopsis and other plant species [9,10]. AS is an important level of regulation in plant gene expression and is involved in a wide range of environmental responses and developmental control [4–7,11]. The functional importance of AS has been demonstrated in sugar signalling [12], development [13], flowering time control [14], light responses [15], dark-light retrograde signalling from chloroplast to nucleus [16], and the circadian clock [17–19].

The circadian clock organises the physiology and behaviour of eukaryotes to optimise their fitness during both day and night [20]. In many crop plants, clock genes have influenced key agricultural traits such as flowering time and yield so that understanding the regulation of the clock itself and of downstream genes is important [21]. In Arabidopsis, the circadian clock controls expression of more than one third of the genes in the genome [22]. The clock consists of a complex network of genes, which are mainly controlled by regulatory feedback loops at the transcriptional, post-translational, and metabolic levels [23–25]. More recently, extensive AS has been identified in core clock genes [17,26,27]. The analysis of the effect of low temperature on AS of core clock genes in Arabidopsis identified changes in AS mainly in *LHY* and *PRR7*, but also *TOC1*, *PRR9*, *PRR5* and *PRR3* [17]. In general, there was an increase in unproductive AS transcripts and a decrease in productive mRNAs. For *LHY*, the change in AS led to a decrease in the level of LHY protein [17]. AS is important in regulating expression of clock genes in other organisms and temperature regulation of AS also occurs in clock genes of *Drosophila* [28,29] and *Neurospora crassa* [30,31].

How the clock modulates its function in different temperatures is a major question in circadian biology. Plants can experience large changes in daily and seasonal temperature but have to maintain clock function and timing. Cold temperatures affect the biochemical properties of most enzymes, including those involved in the circadian clock which can slow down the pace of the circadian rhythm and affect the anticipation responses [32]. The plant clock responds to temperature changes through two mechanisms. Firstly, temperature oscillations entrain the clock and adjusts/corrects its phase, which in turn enables biological activities in the plant to correctly synchronize to diel cycles [33]. Secondly, the plant clock compensates for changes in reaction rates across a wide range of temperatures, and thus maintains a fairly constant pace [32,33]. The identity of the initial mechanism of temperature perception that transduces temperature signals to the circadian clock (also known as plant thermometers) is unknown [34]. Calcium oscillations, as well as phytochromes themselves, may integrate temperature and circadian information [15,32,35]. There is increasing evidence that temperature-associated AS is functionally important in the clock [17,34] and given the increasing association of AS to abiotic responses [6,34,36–38], it is likely that temperature-dependent AS is involved in integrating temperature sensing with downstream processes [34]. AS in the clock may therefore help to synchronise temperature cues with downstream processes in both temperature entrainment and temperature compensation mechanisms [17,34].

The importance of AS in regulating clock function is demonstrated by the effect of mutations in spliceosomal proteins and splicing factors on the period of the clock. For example, mutations in *PRMT5* and *STIPL1* genes in Arabidopsis cause less efficient splicing of *PRR9*, which in turn lengthens the circadian period [19,39]. The splicing factor SKIP regulates the alternative splicing of circadian clock genes in a temperature-sensitive manner, and mutations in *SKIP* lengthen the circadian period [40]. More recently, analyses of a mutation in the GEMIN2 gene has shown that this spliceosomal factor modulates low temperature AS of several clock genes required for proper temperature compensation [18]. Most plant clock studies use Arabidopsis such that our knowledge of the evolution and conservation of AS regulation of circadian clock gene expression in response to temperature is limited. In general, in plants, only a relatively small number of gene-specific or genome-wide cross-species studies have demonstrated conservation of some AS events or behaviour. Gene-specific examples are particular plant serine-arginine rich (SR) protein genes, which have clear conservation of AS splice site sequences [41,42]. Genome-wide AS comparisons have used cDNA, expressed sequence tag (EST) [43], and RNA-seq datasets [9,44] and for example, the clock gene *AtCCA1* has an evolutionarily conserved intron retention event [44]. While conservation of AS events indicates functional relevance, low conservation probably reflects species-specific effects involved in speciation [45].

The circadian clock has a major impact on barley traits [21]. Natural and induced mutations in some barley clock genes have considerably affected flowering time and extended the geographic range where barley is grown [46–49]. There is also evidence that the barley clock contributes to cold resistance [50]. Here, we have identified for the first time alternative splicing events in the barley core clock genes. We show that specific genes, such as the barley orthologues of *AtLHY* and *AtPRR7*, have altered AS when barley seedlings are exposed to low temperature. We demonstrate conservation of AS events and of splicing behaviour of some clock genes in low temperatures in barley and Arabidopsis indicating a similarity of clock regulatory mechanisms. Additionally, we report novel AS events and temperature-sensitive isoform switch which alter the protein-coding sequence of *PPD-H1*, a *PRR7* clock gene orthologue and a major determinant of barley photoperiod response. The analysis of AS in clock genes from different species during the transitions between temperatures may provide valuable information to help understand the role of splicing in temperature entrainment and compensation in the barley clock.

## Materials and Methods

### Plant material

Three barley lines were used in this study: 1) the two-rowed spring barley cultivar Bowman (PI 483237) [51], which contains the naturally occurring monofactorial recessive *Hvprr37_ppd-H1* allele; 2) the Bowman near isogenic line (NIL) *eam1*.*d* (line ID BW281) [52], which is homozygous for the monofactorial dominant *HvPRR37_PPD-H1* gene from KT1031 (a winter barley from Bulgaria, GSHO 1568) and is a BC8 line (8^th^ backcross with Bowman) with a small gene interval, < 1 cM, introgressed on chromosome 2H; and 3) the Bowman NIL *eam8*.*w* (line ID BW290) [52], which is homozygous for the monofactorial recessive *early heading k*/*Hvelf3* gene from Early Russian (a naturally occurring variant, Clho 13839) [53] and is a BC6 line with a 1.5 cM introgressed segment on chromosome 1H. The Bowman *eam1*.*d* line that contains the *HvPRR37_PPD-H1* gene is the effective wild-type genotype and was used for the determination of alternative splicing events.

### Growth conditions

Grains from each line were first germinated in the dark at 4°C on sterile water-soaked filter paper and transferred to small square plastic pots (5 cm^3^) filled with cereal compost mix (225 L Levingtons M2, 225 L Perlite, 0.5 kg Osmocote mini, 2.5 kg Celcote). Each pot contained four to five grains from the same line. Plants were grown in a controlled environment growth cabinet—Microclima 1000 model (Snijders Scientific, The Netherlands)—at 20°C with 16 h light and 8 h dark (Long Day, LD, treatments), unless stated otherwise. To minimise possible microclimate effects, pots with different barley lines and replicates were placed in a randomised array and harvesting was pre-determined in a random order. Illumination of 400 μmol m^−2^ s^−1^ was provided by 12 daylight fluorescent tubes and 3 red/far red fluorescent tubes. Temperature and humidity (70%) were monitored with a data logger (EL-USP-2, Lascar Electronics). No crop protection treatment was applied. To analyse plants at the same stage of development, the majority of the plants were harvested at GS11 of the Zadoks code, when the first leaf is unfolded [54,55]. Plants that were not at this stage at the start of harvest were discarded. Each biological replicate consisted of 4–5 seedlings.

### RNA extraction

Frozen tissue was ground in liquid nitrogen. RNA from up to 100 mg of ground tissue was extracted with the RNAeasy Plant MiniKit (QIAGEN, UK) and RNA preparations were DNase-treated on columns using the RNase-free DNase (QIAGEN, UK), following the manufacturer’s instructions. Each RNA sample was quantified and quality assessed using a Nanodrop^®^ 1000 spectrophotometer (Thermo Fisher Scientific, USA) and further extracted with phenol if required. To confirm that there was no genomic DNA contamination, each RNA sample was subjected to PCR amplification, using primers for *HvPRR59* (S1 Table). DNA-free RNA samples were stored at -80°C.

### cDNA synthesis

cDNA was synthesised from 4 μg of total RNA (20 μL) using the RNA to cDNA EcoDry^™^ Premix (Double Primed) kit (Clontech Laboratories, Takara Biotechnology Company, USA). Reverse transcriptase reactions containing both oligo (dT)_18_ and random hexamer primers were incubated in a thermal cycler (2720 Thermal Cycler, Applied Biosystems^®^, Life Technologies, USA) for 1 h at 42°C, followed by 10 min at 70°C to terminate the reaction. Subsequently, cDNA samples were diluted by adding 100 μL sterile distilled water (SDW) and stored at -20°C.

### Primer design and PCR

Gene-specific primers were designed using the program PrimerQuest from IDT (http://eu.idtdna.com/PrimerQuest/Home/Index) to amplify between 150–1200 bp of genomic sequence (S1 and S2 Tables). Unless stated otherwise, PCR conditions were as follows: PCR was carried out in individual 0.2 mL polypropylene PCR tubes (Axygen^®^, Corning^®^, USA) containing 3 μL cDNA template, 10 μM of each primer, 4 μL of 5 × Green GoTaq^®^ reaction buffer (Promega, USA), 0.2 μL GoTaq^®^ DNA Polymerase (5 U/μL, Promega, USA), 1 μL dNTP (5 mM of each dNTP, Invitrogen, Life Technologies, USA) in a final volume of 20 μL. The PCR programme consisted of an initial 2 min step at 94°C, followed by 25 cycles of: 1) 15 sec at 94°C; 2) 15 sec at 50°C; and 3) 1 min and 30 sec at 72°C, followed by a final extension step of 10 min at 72°C. Negative controls containing RNA template and a positive control containing genomic DNA were subjected to the same procedure to exclude any possible contamination or to detect PCR inhibitors. To analyse PCR amplified fragments, 15 μL of the final PCR product was run on a 1.5% agarose (UltraPure^™^, Invitrogen, Life Technologies, USA), 1 x TBE (50mMTris-Cl pH 8, 50mM Boric Acid, 1mM EDTA) gel.

### High Resolution (HR) RT-PCR

The HR RT-PCR method allows for accurate measurement of multiple alternative splicing events and identification of novel AS events. HR RT-PCR is a co-amplification method based on Simpson et al [56] and has been used previously to validate mRNA quantification, giving good agreement with data from qPCR [17], and RNA-seq data both qualitatively and quantitatively [10,57,58]. For each primer pair, the forward primer was labelled with 6-carboxyfluorescein (FAM). Each HR RT-PCR reaction was run in individual 0.2 mL wells of a 96-well PCR plate (Thermo Fisher Scientific, UK) and usually contained 3 μL of cDNA (template), 20 μM of each primer, 2 μL of 10 × PCR reaction buffer with MgCl_2_ (Roche, UK), 0.25 μL Taq DNA Polymerase (5 U/μL, Roche, UK), 1 μL dNTP (5 mM of each dNTP, Invitrogen, Life Technologies, USA) in a final volume of 20 μL. The PCR programme included an initial 2 min step at 94°C, followed by 20–28 cycles of: 1) 15 sec at 94°C; 2) 15 sec at 50°C; and 3) 1 min and 30 sec at 70°C [59] with a final extension step of 5 min at 70°C. The majority of PCR reactions here were carried out for 24 cycles [59]. In some cases, due to variation in transcript abundance of some of the AS transcripts, PCR cycle number was between 20 and 28 cycles (for example, highly expressed genes were amplified with 20 cycles) [59]. Subsequently, 1 μL of the resultant PCR reaction was mixed with 8.5 μL of Hi-Di^™^ formamide (Applied Biosystems, USA) and 0.05 μL of GeneScan^™^ 500 or 1200 LIZ^®^ standard (Applied Biosystems^®^, Life Technologies, UK) and loaded on the ABI 3730 automatic DNA sequencer (Applied Biosystems^®^, Life Technologies, UK). PCR products were accurately sized, to single base pair resolution, and quantified in Relative Fluorescent Units (RFU), which are the peak areas from spectral data (proportional to the amount of total product present), calculated by GeneMapper software (version 3.7, Applied Biosystems^®^, Life Technologies, USA). To compare transcript data, variation arising from unequal quantities of starting RNA was removed by normalising clock transcript RFU to the arithmetic mean RFU values of fully spliced *HvUBC21* and *HvPP2AA2* control transcripts.

### AS discovery in barley clock genes

To identify AS isoforms, BLAST searches were performed using barley clock genomic sequences identified previously [60] against GenBank and barley RNA-seq transcript databases [61]. For the RNA-seq data set, AS discovery analyses were carried out using the TABLET program version 1.14.10_20 [62]. To detect novel AS events and determine the sequences of unknown alternatively spliced isoforms, RT-PCR and cloning of amplified products was carried out. Sequences were analysed using Sequencher software version 4.9 (Gene Codes Corporation), which allowed trimming and cleaning of the sequences before alignment using the ClustalOmega software [63] on to genomic contigs to identify alternative splicing events and define the splice sites used.

### Analyses of AS in clock genes in response to temperature changes

To observe the behaviour of clock gene expression and AS to changes in temperature, leaf samples of 1-week old plants, grown at 20°C, were collected at 2.5 h after dawn (S1 Fig). This time-point corresponds to the peak of expression of *HvLHY*. At dusk, 15.5 h after the first harvesting, the temperature of the cabinet was reduced to 4°C, and this temperature was maintained, along with LD condition, for four days. Additional leaf samples were harvested at 2.5 h after dawn on days 1, 2 and 4 after transfer to 4°C (S1 Fig). At dawn of the 5th day at 4°C, the temperature was increased from 4°C to 20°C. Another two sampling points took place at 2.5 h after dawn on days 1 and 2 after adjustment to 20°C. The cabinet took less than 90 min to decrease the temperature from 20°C to 4°C and vice-versa. A minimum of three biological replicates were taken per time point, each consisting of one or two leaves from at least three independent plants from a single pot. Pots were placed in a random array and harvesting carried out in a random order. Leaf material was rapidly frozen in liquid nitrogen and stored at -80°C.

### Cycloheximide treatment for analysis of nonsense-mediated decay

Primary leaves were harvested at dusk and the epidermis was stripped with fine forceps from portions of the abaxial leaf surface, exposing mesophyll over l/3 to 1/2 of leaf surface [64]. Four to eight segments 0.5 to 1 cm long were cut from each leaf, avoiding the 2 cm at the leaf tip and base, as well as poorly stripped areas. Four to five segments from different leaves were floated (stripped surface down) on 2 ml of MS media in wells of a 12-well cell culture plate (Costar, Fisher, USA). Cycloheximide (CHX) stock solution (20 mg/ml), diluted in absolute ethanol, was added to the MS media to a final concentration of 350 μM. Negative controls were prepared by adding the same volume of absolute ethanol to the liquid MS media. Plates containing leaf segments were then incubated in the controlled environmental cabinet at 20°C or, in parallel, transferred to 4°C. Leaf harvesting, stripping of the epidermis and beginning of incubation were performed in approximately 30 minutes. Light-dark conditions were maintained throughout the experiment. A minimum of three biological replicates were collected at 2.5 h after dawn, totalling approximately 10 h of incubation in CHX.

### Statistical tests

To analyse expression and AS data, sets of data were first tested using the Shapiro-Wilk test for Normality. Normally distributed data sets were analysed using an Analysis of Variance (ANOVA). The associated *P* value calculated in the ANOVA test is two-tailed and a significance level of 5% (α = 0.05) was used. GenStat (15^th^ Edition version 15.1.0.9425, VSN International LTD) was used for all statistical analysis.

## Results

### Identification of AS isoforms of barley clock genes

Arabidopsis clock genes show extensive AS and the effects of AS on clock functions have been demonstrated [17,19,39,44,65]. To carry out a systematic identification of AS isoforms of barley core clock genes and assess splicing behaviour, homologues of Arabidopsis clock and clock-associated genes were first identified in barley using an *in silico* approach [60]. This approach identified both genomic and cDNA sequences of barley clock genes, which were used to annotate and determine gene structure (intron/exon) and develop gene models. Of the barley clock homologues [60] *HvFT1*, *HvFT2*, *HvLHY*, *HvCO1*, *HvCO2*, *HvTOC1*, *HvGI*, *HvELF3*, *HvFKF1*, *HvGRP7a*, *HvGRP7b*, *HvZTLa*, *HvZTLb*, *HvELF4-like3*, *HvPRR37*, *HvPRR73*, *HvPRR59* and *HvPRR95* contained introns while *HvCABa*, *HvLUX* and *HvELF4-likeA* did not.

To identify AS events in these barley clock genes, firstly, publicly available EST and cDNA sequences as well as the extensive barley RNA-seq data [61] were aligned to barley genomic sequences and, secondly, a systematic standard RT-PCR was carried out to detect alternative transcripts experimentally. RT-PCR was performed on RNA from plants grown in 16h light/8h dark at 20°C and, in parallel, 8h light/16h dark at 4°C and harvested every three hours over a 24 h period. The primers used for AS discovery for the subset of genes chosen are shown in S1 and S2 Tables. Alternative transcripts were characterised by cloning and sequencing or, in some cases, were predicted (especially for IR events) based on expected product size, intron sequence and presence of potential alternative splice sites in amplified regions.

Extensive AS was identified with 51 novel AS events being detected in the set of twelve clock genes (Table 1, S1 File, S3 Table). Having identified putative AS events in these barley clock genes we next designed fluorescently-labelled primer pairs to the relevant regions to perform high resolution (HR) RT-PCR. HR RT-PCR allows the accurate and simultaneous detection and sizing of RT-PCR products representing AS events and their relative abundance (see Material and Methods). The AS events identified were either unproductive by virtue of disrupting the coding region and introducing premature termination codons or were in frame and could increase proteome diversity. Many of the AS events were IR events (29/51), of which 19 were found in low abundance (< 2% of total transcripts) in all conditions analysed here and probably represent partially spliced or unspliced transcripts [66]. The AS events generated 71 different transcripts, including the 12 normally spliced transcripts. The abundance of the 59 alternatively spliced transcripts varied extensively, with twelve having levels of between 11–76% of the total transcripts in WT plants grown at 20°C or 4°C. These transcripts were found in four different genes: *HvLHY*, *HvPRR37*, *HvGI* and *HvCO2* (Table 1, described below). No alternative transcript isoforms were detected for *HvFT1*, *HvFT2* or *HvCO1*. Alternatively spliced transcripts of *HvTOC1*, *HvELF3*, *HvGRP7a/b*, *HvPRR95* and *HvPRR59* were identified but were in low abundance representing <2% of the total transcripts at 20°C (S1 File, S2 Fig, S3 Table). Nevertheless, conservation of some AS events was observed between Arabidopsis and barley (Table 2). It should be noted that although many AS transcripts are found in low abundance here, some of these events may occur more frequently in other growth conditions.

**Table 1:** Abundant AS events in barley core clock and clock-associated genes.

Gene	AS events	% total transcript	Observation
*LHY*	Various IR at the 5’UTR (IR 1, 2 and 3)	Together, they form up to 26% total transcripts at 4°C;	These add up to 12 uAUG (up to 31 aa uORF)
	Alt 3' ss Intron 1	12% total transcripts at 4°C;	Adds 1 uAUG (31 aa uORF)
	Intron 4 retained (I4R)	40% total transcripts at 4°C	Creates PTC on Exon 5
	Intron 8 retained (I8R)	37% total transcripts at 4°C	Altered C-terminus coding region
*PRR37*	Cryptic intron E1 (-102 nt)	20% total transcripts at 20°C	Loss of uORF coding for 48 amino acids
	Intron 1 retained	17% total transcripts at 20°C	Adds uORF coding for 45 amino acids that overlaps functional ORF by 35 nt
	alt 3' ss E6 (-6 nt)	33% total transcripts at 20°C	Altered protein coding sequence
	alt 5' ss E6 (- 45 nt) and alt 3' ss E6 (-6 nt)	24% total transcripts at 20°C	Altered protein coding sequence
	alt 5' ss E6 (- 45 nt)	76% total transcripts at 4°C	Altered protein coding sequence
*GI*	E2 skipping and Alt 3’ ss I2 (+12 nt)	Up to 30% total transcripts at 20°C	Removes 3 uAUG (up to 18 aa uORF)
	Alt 3’ ss I2 (+12 nt)	Up to 22% total transcripts at 20°C	-
	alt 5’ ss E2 (-8 nt) and alt 3’ ss I2 (+12 nt)	Up to 11% total transcripts at 20°C;	-
*CO2*	Alt 3’ ss (+68 nt)	35% total transcripts at 20°C	Creates PTC

**Table 2:** Conservation of AS events in barley core clock genes in Arabidopsis.

Gene	AS events in barley	AS events in Arabidopsis	Type of AS conservation
*LHY*	Various IR at the 5’UTR (IR 1, 2 and 3) and I4R	I1R in Arabidopsis	Conserved AS behaviour
	Alt exon 6a	Alt exon E5a in Arabidopsis	AS behaviour and AS region conserved
*PRR37* (*PRR7*)	alt 3' ss E6 (-6 nt)	Only in monocots ^b^	AS event conserved
	alt 5' ss E6 (- 45 nt)	Only in monocots ^b^	AS event conserved
	CrIn E8 (-249 nt)	CrIn E8 (-84 nt) in Arabidopsis	AS region conserved
*GI*	I14R	I13R in Arabidopsis	AS event conserved
	I15R	I14R in Arabidopsis	AS event conserved
*TOC1* ^a^	I1R	I1R in Arabidopsis	AS event conserved
	I3R	I3R in Arabidopsis	AS event conserved
	I3R and I4R	I3R and I4R in Arabidopsis	AS event conserved
*PRR5/9* ^a^	I2R (HvPRR95) I3R (HvPRR59)	I3R in PRR9	AS event conserved

^a^ More info in Supporting Information.

^b^ Not seen in Arabidopsis but conserved in monocot grasses.

#### HvLHY

**Fig 1:**
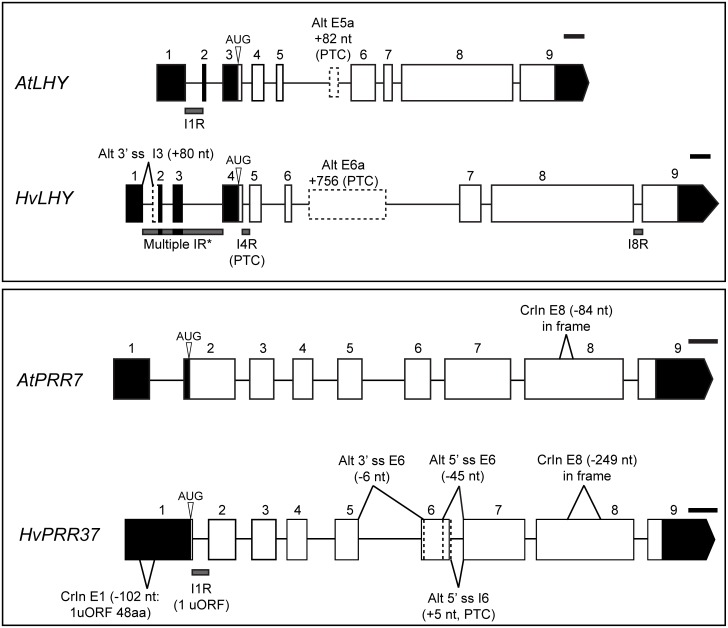
Genomic structure and conserved AS events of Arabidopsis and barley *LHY* and *PRR37*/*PRR7*. In the Arabidopsis genes, only those AS events which show conservation to barley are shown—for other Arabidopsis clock genes AS events see James et al. (2012a). Other abundant AS events in barley are also shown. Exons are numbered; 5’ and 3’ UTRs are dark boxes; coding sequences are open boxes. Alt, alternative; ss, splice site; I, intron; R, retention; E, exon; CrIn, cryptic intron; AUG, translation start site. Small black lines on the top right of each gene structure represent a scale of 200 bases. *, multiple intron retention events in the *LHY* 5’UTR are explained in the text.

Of the ten AS events observed for barley *LHY* the most abundant AS event at 4°C was retention of intron 4, which is the first intron in the coding sequence lying upstream of exons 5 and 6 which encode the MYB domain (Table 1, Fig 1). A second abundant AS event at 4°C was retention of intron 8 which would affect the C-terminal sequence of the protein (Table 1; Fig 1). Neither of these AS events were observed in Arabidopsis. Interestingly, the long intron 6 of *HvLHY* contained an alternative exon, E6a, of 756 nt which contained premature termination codons (PTC). In Arabidopsis, the corresponding long intron (intron 5) also encodes a PTC-containing alternative exon (E5a) which is included at low temperature generating an unproductive transcript (Fig 1, Table 2) [17]. Although the sequences of the alternative exons are different, their increased inclusion at low-temperature (see below) and that neither can encode LHY protein illustrates conservation of AS behaviour. Finally, the *HvLHY* gene has a complex 5’ UTR (4 exons and 3 introns) and combinations of retention of the three introns together made up to 26% of *LHY* transcripts at 4°C (Table 1). Retention of intron 1 in Arabidopsis is an important low-temperature induced event which regulates LHY levels [17]. Although this represents conservation of AS behaviour, due to the complexity of the barley *LHY* 5’UTR, it is not possible to directly equate specific AS events.

#### *HvPRR37* and *HvPRR73*

*HvPRR37* retention of intron 1 (I1R) was mostly abundant at 20°C and it created an uORF that codes for 45 amino acids that overlaps the functional ORF by 35 nt. This arrangement of uORF and ORF has been shown to i) trigger nonsense-mediated decay (NMD) [66] and ii) to repress downstream CDS translation [67]. In addition, two alternative splice sites generated abundant, novel *HvPRR37* protein-coding mRNA isoforms: 1) an alternative 3’ splice site which removes 6 nt at the 5’ end of exon 6 (alt 3’ss E6) and/or 2) an alternative 5’ splice site that removes 45 nt from the 3’ end of exon 6 (alt 5’ss E6 -45nt) (involving 2 and 15 amino acids, respectively). These AS events were not seen in the Arabidopsis *PRR7* orthologue but a BLASTn search of the GenBank monocot EST database using exons 5–7 of *HvPRR37* identified transcripts with an equivalent alt 5’ss E6–45 nt in *Leymus* spp. (Triticeae) [68] and an alt 3’ ss E6 (removing 6 nt) in *Oryza* spp. [69,70]. In addition, a nucleotide alignment of *HvPPD-H1* E6 with *PRR7*, *PRR3*, *PRR37* and *PRR73* genes from three dicots and six monocots suggested that both AS events for the *PRR37* gene were conserved among monocots only (S2 Fig). Similarity in AS events in the putative orthologues *AtPRR7* and *HvPRR37* was seen in both having an in frame cryptic intron in E8 that removes 84 nt or 249 nt (28 and 83 amino acids) respectively (Fig 1). Interestingly, *AtPRR3* and *HvPRR73* undergo two similar AS events, alt 5’ ss E2 (-7 nt) and I4R, even though these genes are not orthologues.

#### HvGI

*HvGI* showed three abundant AS events in the 5’ UTR, two of which resulted in upstream open reading frames (uORFs) of up to 26 amino acids (Table 1; S3 Fig and S3 Table). Similar AS events were not found in Arabidopsis. *GI* transcripts with alternative 5’ UTRs represented up to 75% of total transcripts. In the *HvGI* coding region, three intron retention events involving I13R, I14R and I15R were observed. Retention of introns 14 and 15 was conserved in Arabidopsis with the *AtGI* orthologous introns 12 and 13 showing retention but all of these IR events were of low abundance in both barley and Arabidopsis [17].

#### HvCO2

*HvCO2* has a single intron and transcripts with an alt 3’ss in the intron, which added 68 nt and introduced a PTC, made up around 35% of transcripts at 20°C (Table 1, S3 Fig). This AS event was not conserved in the Arabidopsis homologues *CO* and *COL1*.

### NMD-sensitivity of unproductive AS transcripts

One of the features of the low temperature AS response of Arabidopsis clock genes is the generation of unproductive mRNA transcripts, some of which are degraded by NMD [17]. To demonstrate NMD and assess the impact of unproductive transcripts, NMD-impaired Arabidopsis mutants *upframe shift* (*upf*) *1* and *3* have been analysed and increased abundance of NMD-sensitive transcripts demonstrated [17,66]. No such mutants exist in barley, and we, therefore, had to establish a system to suppress NMD. We developed an assay where barley leaf material was exposed to cycloheximide (CHX). NMD is translation-dependent and as CHX blocks translation, NMD-sensitive transcripts should increase in abundance [66].

**Fig 2:**
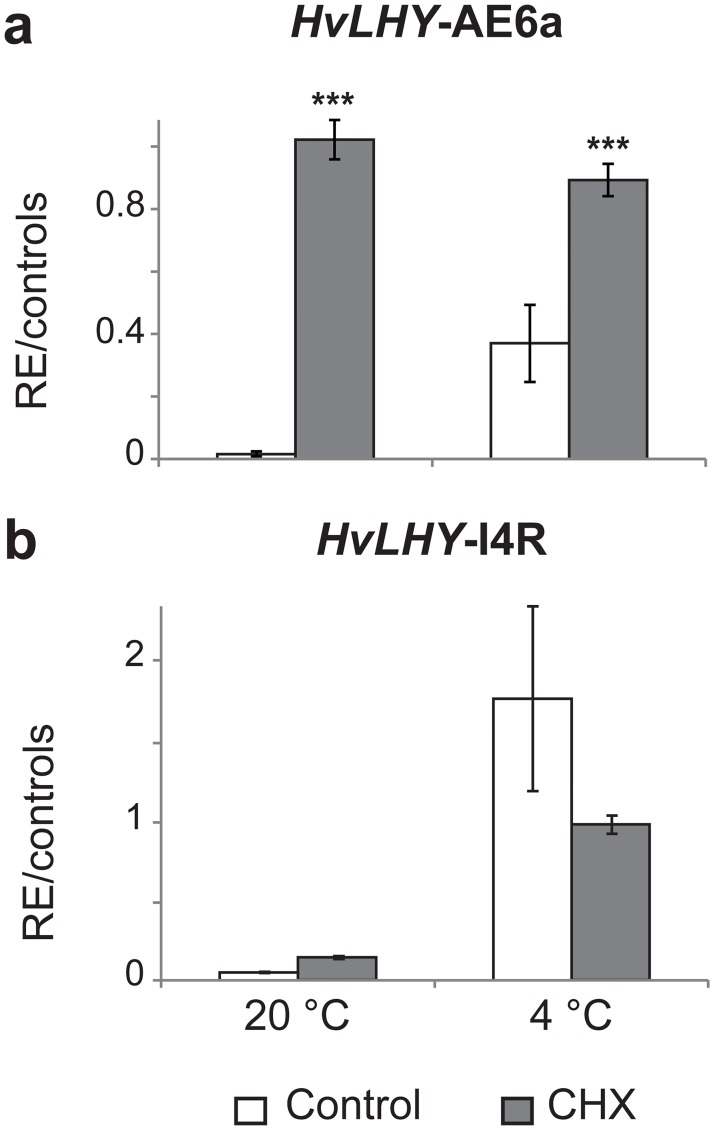
NMD sensitivity of alternatively spliced transcripts of *HvLHY*. The relative abundance of different transcripts was measured in control (white) and CHX (grey) treatment conditions at 20°C and 4°C by high resolution RT-PCR; n = 3. RE, relative expression. (a) *LHY* transcripts containing Exon 6a showing significantly increased abundance in CHX treatment at both temperature conditions. *** *P* < 0.001. (b) *LHY* transcripts containing I4R showing no increase in abundance in CHX-treated material at both temperature conditions.

To establish the assay we used the Bowman NIL *eam8*.*w* (line ID BW290) [52], which is homozygous for the recessive *early heading k*/*Hvelf3* mutant allele which contains a PTC and acted as an endogenous control (S4 Fig). The *Hvelf3* PTC-containing transcript increased significantly in the *Hvelf3* mutant while no increase was observed in the WT or Bowman lines which contained the wild-type *HvELF3* transcript (S4 Fig). HR RT-PCR was performed on CHX-treated stripped leaf material using primers specific to the *HvLHY* and *HvPPD-H1* alternative transcripts. A clear increase in the AS transcript of *LHY* E6a was found in CHX-treated material at both 20°C and 4°C samples, suggesting that this AS event is NMD-sensitive (Fig 2a). No other AS event in *LHY* and *PPD-H1* showed significant NMD-sensitivity in 20°C and 4°C conditions (e.g. Fig 2b).

### Dynamic changes in expression and AS of *HvLHY* and *HvPPD-H1* under low temperature

The Arabidopsis clock genes that showed the greatest effect on AS at low temperature were *AtLHY* and *AtPRR7* [17]. Having defined the AS events of clock genes in barley, we investigated dynamic changes in AS and whether transient or longer term low temperature conditions influenced AS of the barley orthologues, *HvLHY* and *HvPRR37/PPD-H1* and its paralogue *HvPRR73*. The HR RT-PCR system was used to examine the relative levels of fully-spliced (FS) transcripts versus transcripts containing different AS events identified for these genes. Leaf samples of barley lines grown in LD conditions were collected at 2.5 h after dawn (the time of peak expression of *HvLHY* and *HvPRR37* at 20°C) in different temperature conditions: 20°C prior to transfer to 4°C; days 1, 2 and 4 at 4°C; day 1 at 20°C following return of plants to 20°C and day 2 at 20°C (S1 Fig).

#### Reduced expression and increased AS of *HvLHY* at low temperatures

**Fig 3:**
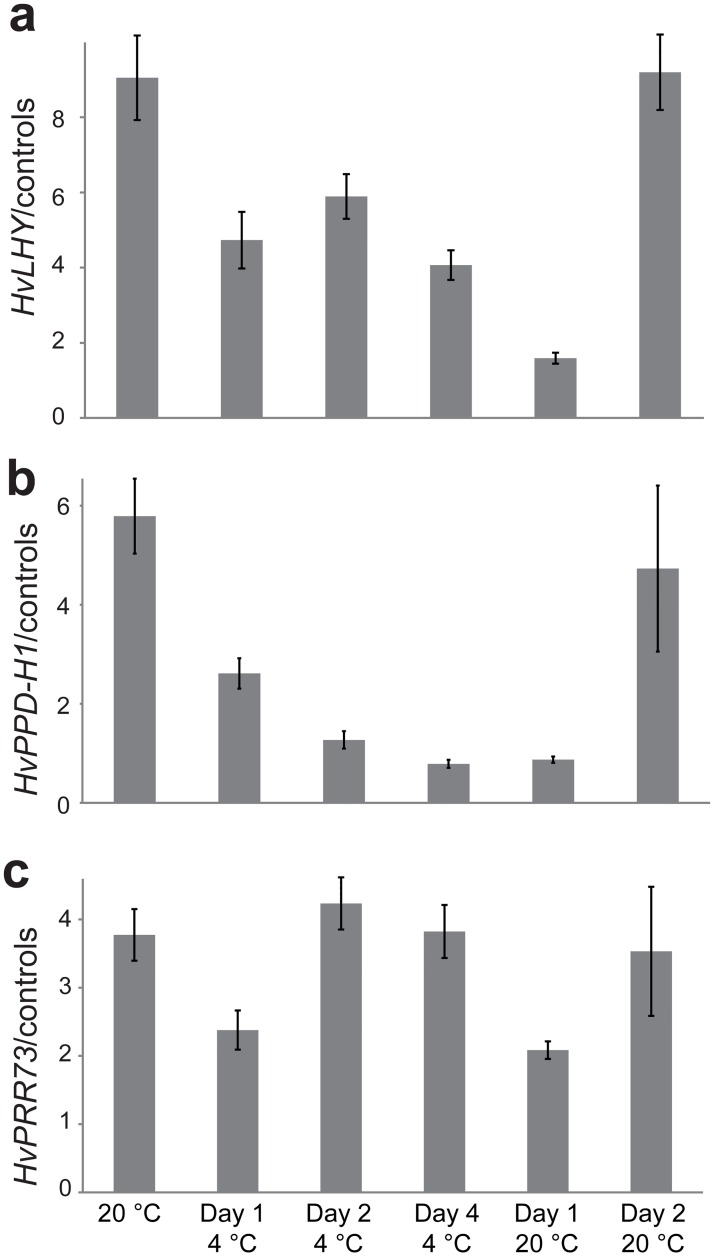
Expression changes of clock genes during transfer from 20°C to 4°C. Total transcript levels of (a) *HvLHY*, (b) *HvPPD-H1* and (c) *HvPRR73* in the morning (2.5 h after dawn) at 6 different time-points: 20°C, Day 1 at 4°C, Day 2 at 4°C, Day 4 at 4°C, Day 1 at 20°C and Day 2 at 20°C (see S1 Fig). Results obtained with different HR RT-PCR primer pairs were considered as technical replicates. Error bars: SEM from three biological replicates. The *LHY* and *PPD-H1* levels on Day 1 at 20°C were low and probably reflected that this time point represents effectively one hour after switching to 20°C from 4°C, showing that recovery of transcript levels takes longer than one hour.

**Fig 4:**
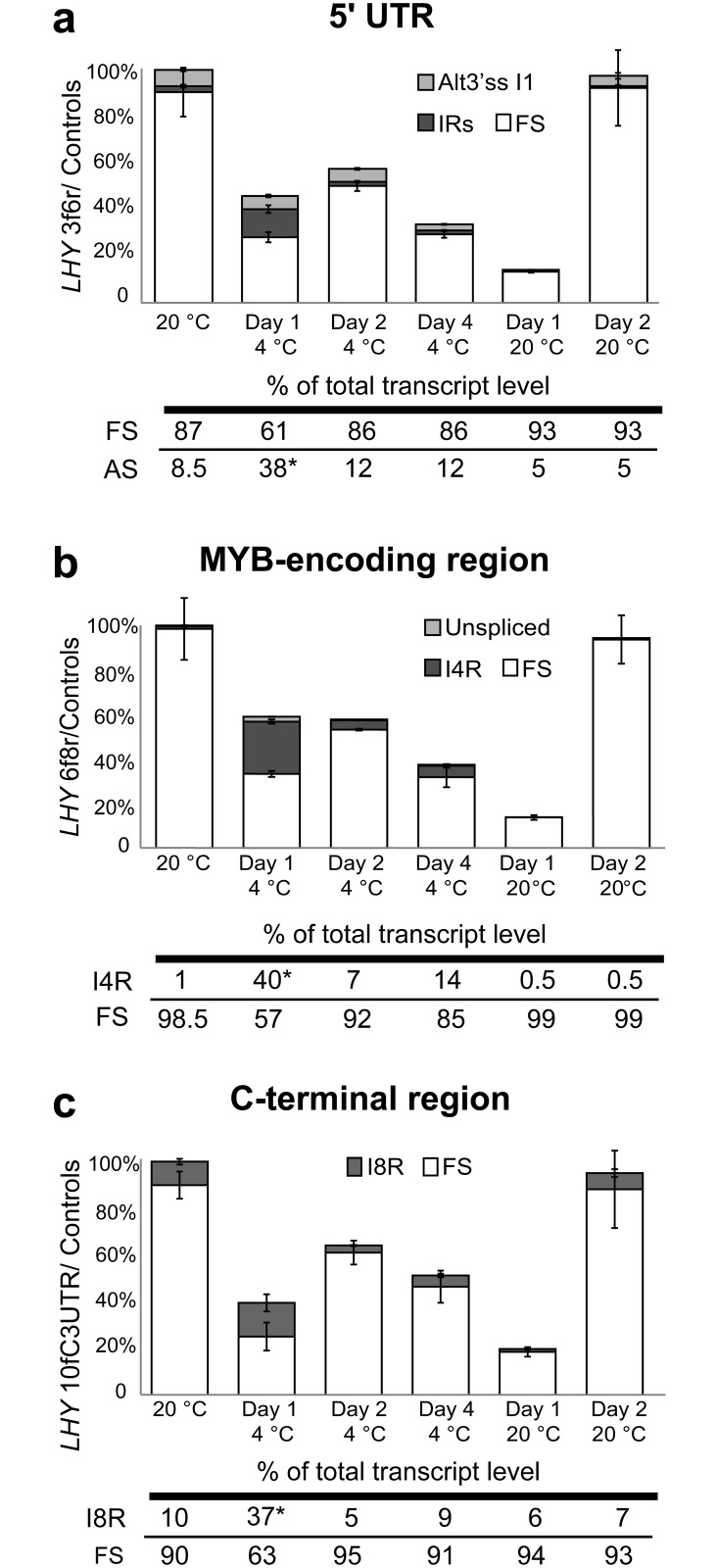
Relative abundances of *LHY* FS and AS transcripts during temperature changes. WT plants were assessed using HR RT-PCR primers spanning (a) the 5’ UTR region, (b) the MYB-encoding region and (c) the C-terminal coding region. Error bars are SEM of three biological replicates. In (a), the IRs fraction is made up of multiple transcripts containing intron retention: I2R & I3R, I3R & Alt3’ss I1, I3R, I1R & I2R, I1R, and I2R. The levels of total transcripts at the different time-points are shown by the height of the histogram bars relative to the 20°C value (100%). The relative amounts of AS variant transcripts are illustrated as a proportion of these totals. Relative expression values of abundant AS events are shown in the tables below the histograms with significantly different AS/FS ratio compared to 20°C data shown with an asterisk (*P* < 0.01). FS—fully spliced; InR—intron retention of intron n.

Changes in total *LHY* transcript levels were observed during temperature transition experiments in WT plants (Fig 3a). *LHY* mRNA levels decreased from 100% at 20°C to 40–60% on Days 1, 2 and 4 at 4°C. On Day 2 following the return to 20°C, *LHY* transcript levels increased four to six times over the 4°C (Day 4) levels to return to the original 20°C levels. Using HR RT-PCR primers spanning the 5’UTR at 20°C the majority of transcripts were fully spliced and a number of AS events were detectable but together made up only 8.5% of the total. Twelve hours after transfer to 4°C (Day 1), total transcript levels were reduced to 42% of the level at 20°C, the level of fully spliced transcripts reduced substantially while AS transcripts increased (Fig 4a). The combined effect resulted in *HvLHY* fully spliced transcripts being reduced to approximately 25% of the level at 20°C. The AS events which increased in abundance were retention of *HvLHY* introns 1, 2 and 3 (adding 2, 3 and 7 uORFs, respectively) and an alternative 3’ splice site at intron 1 (adding 1 uORF). It is not possible to predict whether these low temperature-induced AS events affect LHY protein production as seen is Arabidopsis for intron 1 retention [17]. Following the first day of the transition from 20°C to 4°C, levels of these events reduced and although levels of fully spliced transcripts remained lower than at 20°C, the relative levels to AS events remained stable at 12% (Fig 4a).

HR RT-PCR of the regions encoding the *LHY* MYB domain and the C-terminus coding regions showed a striking retention of *LHY* intron 4 (I4R) and 8 (I8R) during the transition from 20°C to 4°C (Fig 4b and 4c). I4R (*P* < 0.001) and I8R (*P* < 0.01) significantly increased in both absolute terms and as a proportion of total *LHY* transcripts, but decreased considerably over the following days at 4°C. I4R changed from around 1% of total transcripts at 20°C to 40% during the first day after reduction in temperature and 14% after acclimation to 4°C. I8R increased from around 10% of total transcripts at 20°C to 37% during the first day at 4°C and 9% after acclimation to 4°C. Although retention of *LHY* intron 4 and 8 create PTCs they were not expected to be NMD-sensitive [10,66] and plants treated with CHX did not have significantly higher levels of I4R (Fig 2b).

In Arabidopsis, an important low temperature-dependent AS event is the inclusion of an alternative exon (E5a), in the long intron 5 which continues to accumulate with exposure to 4°C [17]. The equivalent intron in *HvLHY* is intron 6. At 20°C, a single product representing the fully spliced transcript (FS) was observed using HR RT-PCR primers spanning *LHY* exons 6 and 7. Plants acclimated to 4°C (Day 4) expressed very low levels (< 0.7% of total transcripts) of an AS transcript with a cold-specific alternative exon E6a (Fig 1; data not shown). To obtain a clearer indication of when alternative E6a is included, RT-PCR was carried out using primers located in E6 (forward) and E6a (reverse) (S5 Fig). The proportion of *LHY* transcripts specifically containing E6a increased upon cooling to 4°C and continued to be detectable while the plants were maintained at 4°C, but decreased when plants were returned to 20°C (S5 Fig). The E6a-containing transcripts are NMD-sensitive transcripts as their abundance increases in the CHX treatment (Fig 2a). Taken together, at low temperatures, the overall levels of *LHY* transcripts decrease and the proportion of cold-dependent AS isoforms increase, which contributes to a substantial reduction in functional *LHY* mRNA levels, likely to lead to a decrease in functional LHY protein. This splicing behaviour is consistent with that observed in Arabidopsis *LHY* [17].

#### An isoform switch decreases diversity of protein-coding transcript isoforms of *HvPPD-H1/HvPRR37* at low temperatures

**Fig 5:**
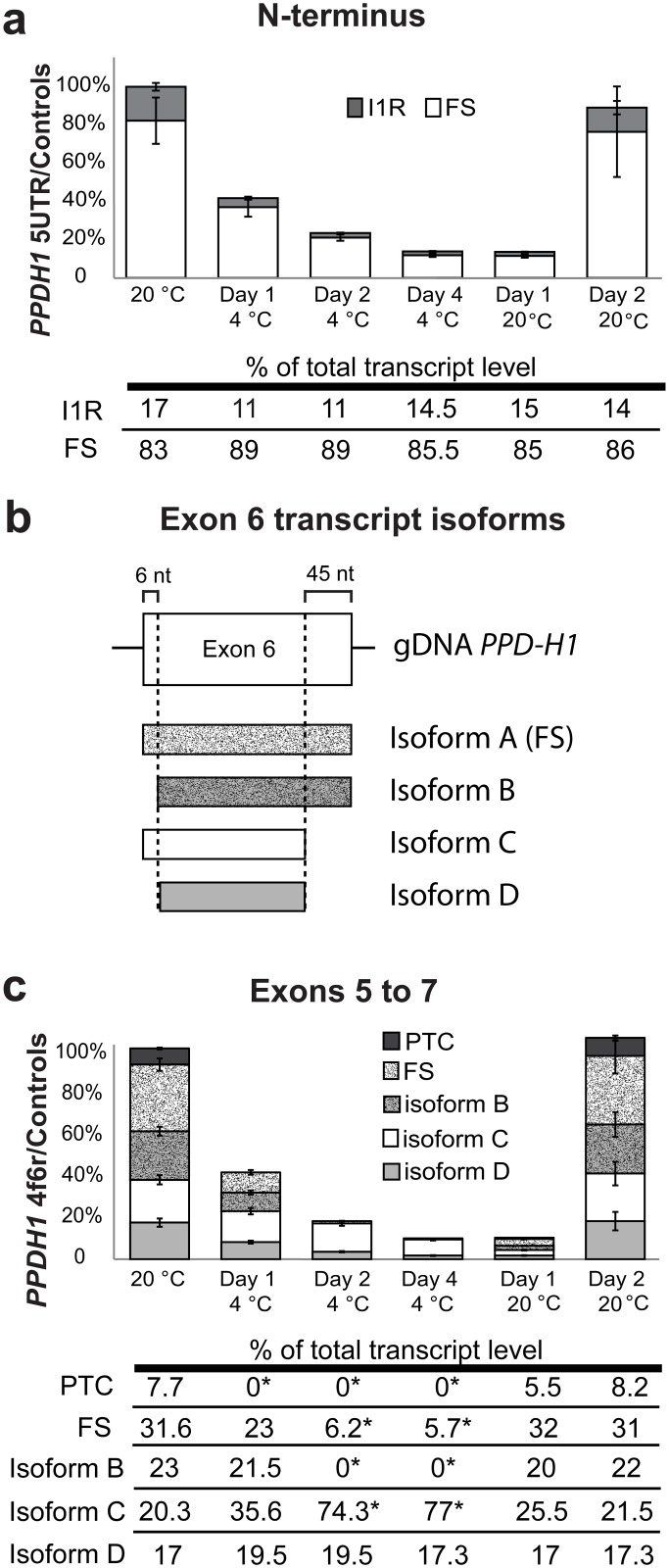
*PPD-H1* AS during temperature changes. (a) WT plants assessed using HR RT-PCR primers spanning the N-terminus region. I1R –intron 1 retention. (b) *PPD-H1* exon 6 undergoes AS to produce four different protein-coding isoforms: A, the fully spliced (FS) transcript; B, transcripts with only the Alt3’ss E6 (-6 nt); C, transcripts with only the Alt5’ss E6 (-45 nt); and D, transcripts with both Alt3’ss E6 (-6 nt) and Alt5’ss E6 (-45 nt). (c) WT plants assessed using HR RT-PCR primers spanning the region between exons 5 and 7. To simplify histograms, the PTC fraction is made up of multiple transcripts containing: I6R, I6R & Alt3’ss E6 (-6 nt), and Alt5’ss E6 (+5 nt). Error bars are SEM of three biological replicates. The levels of total transcripts at the different time-points are shown by the height of the histogram bars relative to the 20°C value (100%). The levels of AS variant transcripts are illustrated as a proportion of these totals. Relative expression values of abundant AS events are shown in tables below the histograms with significantly different AS/FS ratio compared to 20°C data shown with an asterisk (P < 0.01).

*PPD-H1* mRNA levels in WT plants decreased from 100% at 20°C to 45% on Day 1 at 4°C and 13% on Day 4 at 4°C (Fig 3b). On Day 2 after the return to 20°C, *PPD-H1* transcript levels increased to almost their original 20°C levels showing a rapid response to temperature decrease and increase. HR RT-PCR across the N-terminal coding region (Fig 5a) or exons 2–5 (data not shown) did not show cold-dependent AS events. The major changes in *HvPPD-H1* AS upon exposure to low temperature were found for the two in frame AS events affecting exon 6. An alternative 5’ splice site in exon 6 removed 45 nt (alt 5’ ss E6 -45nt) and an alternative 3’ splice site in exon 6 removed 6 nt (alt 3’ ss E6 -6nt) from the 3’ and 5’ ends of exon 6 respectively (Fig 5b). At 20°C, the most abundant transcript is isoform A (called fully spliced—FS) present at 31.6% of total transcript (Fig 5b and 5c). At low temperatures, we observe an isoform switch: the levels of the FS isoform A and isoform B (alt 3’ ss E6 -6nt) are decreased significantly from around 54.7% of total transcripts at 20°C to 5.7% after acclimation to 4°C, whereas the levels of isoform C (alt 5’ ss E6 -45nt) is significantly increased from around 20.3% of total transcripts at 20°C to 77% after acclimation to 4°C (Fig 5b and 5c). The relative amount of isoform D does not change at different temperatures. Interestingly, on Day 1 at 20°C, which was effectively 1 hour at 20°C after transfer from 4°C, isoforms A and B increased such that the proportions of the different isoforms returned to the original 20°C levels. This shows a much faster isoform switch in response to increased temperature, suggesting that that change in AS is due to temperature-dependent AS regulation rather than transcriptional regulation. In summary, changes in temperature can induce dynamic changes in AS to alter levels of different AS isoforms. For *HvPPD-H1*, we observed changes in the diversity of protein-coding transcript isoforms of at the transcriptional and/or AS levels. Although the use of in-frame alternative splice sites changed drastically at low temperatures, it was not possible to predict any potential function of the change in protein-coding sequences as no protein domain was found in this region of the protein. Other low abundance unproductive AS events in *PDD-H1* are reported in S1 File. It is also noteworthy that the paralogues *PPD-H1* and *PRR73* behaved very differently in their AS response to low temperature (Fig 3, S1 File).

### Dynamic low temperature changes on expression of clock genes in clock mutants

The effects of temperature changes on *HvLHY*, *HvPPD-H1* and *HvPRR73* expression and AS patterns were tested in the three barley lines (Bowman *Hvprr37_ppd-H1*, BW281 *HvPRR37_PPD-H1* and BW290 *early heading k/Hvelf3*) using different HR RT-PCR primer pairs (S1 and S2 Tables) which spanned most of the gene sequences. Expression of *LHY*, *PPD-H1* and *PRR73* varied significantly among the three different lines across the low-temperature time course reflecting the influence of these mutations on expression of the core clock genes (S6a and S6b Fig). For example, *Hvelf3* plants had significantly higher levels of *ppd-h1* when compared to WT *PPD-H1* expression at 4°C, which suggests that under low temperature conditions, the *elf3* mutation has a stronger effect on *PPD-H1* expression (S6b Fig). In addition, *HvLHY* expression was significantly lower in the *Hvelf3* mutant at 20°C (S6a Fig) while expression of *HvPRR73* was similar across all three lines (S6c Fig). In contrast, AS results for the three barley lines were virtually identical (data not shown), indicating that the *ppd-H1* and *elf3* mutations have no direct effect on *LHY*, *PPD-H1* and *PRR73* AS. Therefore only the results for the BW281 (effective wild-type) are shown and representative HR RT-PCR electropherograms illustrate the clear differences in transcript isoform levels between the time points (e.g. *HvLHY* in S7 Fig). For the control genes *HvUBC21* and *HvPPDAA2*, and the clock genes themselves, the majority of introns were spliced efficiently at both 20°C and 4°C and the relative abundance of many transcripts containing IR events was not affected by cooling (data not shown). Therefore, transient AS events seen upon cooling were not caused by a non-specific and general decline in the splicing activity under these conditions, but rather, represent subtle and specific modulation of mRNA diversity and expression levels.

## Discussion

We have identified 51 AS events (in 59 AS transcripts) in barley core clock and clock-related genes at a system-wide level. Similar to Arabidopsis [17], many transcripts were low abundance transcripts and many were IR transcripts, the majority of which were also in low abundance. Nevertheless, eight AS events were significantly modulated by transient and long-term low temperature changes and may play a significant role in regulating the expression of their cognate clock genes and the response of the plant circadian clock to temperature. In both barley and Arabidopsis, the majority of AS events introduced PTCs leading to non-productive mRNAs [17]. However, the abundant AS events in barley *PPD-H1* (Fig 5b and 5c) and *LHY* (I8R) modulate the levels of different protein-coding isoforms. In particular, the AS events in *HvPPD-H1* undergo rapid isoform switches in response to both decrease and increase in temperature. Alternative protein isoforms are particularly interesting for LHY and PPD-H1/PRR37, since they can form homo- and hetero-dimers [71] and the different combinations formed between different AS protein isoforms may have different properties, such as activity, protein-protein interactions and downstream targets. Further studies of these different protein isoforms may help understanding their role in maintaining clock function at low temperature.

The role of AS in the regulation of gene expression and AS in response to environmental changes such as temperature has started to be defined for some plant clock genes. For example, microarray studies in Arabidopsis showed that at low temperatures, the levels of *LHY* decreased to 50% of their normal levels. Analysis of AS showed that two-thirds of these transcripts were likely to be unproductive such that the level of *AtLHY* in response to cold was reduced to 15–20% of normal (20°C) levels [17]. This AS effect was only revealed by transcript level resolution. Furthermore, many cold-induced AS changes in plant clock genes generate unproductive transcripts, some of which may be NMD-sensitive. We also developed a new NMD assay for barley using cycloheximide and showed NMD-sensitivity for the *HvLHY* E6a-containing transcript. These results illustrate the importance of analysing gene expression, in general, and clock genes in particular at the AS/transcript level.

Decreasing temperature causes reduction in the expression peak of functional *AtLHY* and an increase in non-productive AS transcripts containing I1R (in the 5’UTR) and E5a (present in the long intron immediately after MYB-encoding region), which were sensitive to NMD [17,72]. It was also shown that transcription of *AtLHY* was not altered in the cold and the observed reduction in AtLHY protein was therefore due to AS [17]. Barley *LHY* showed the same AS behaviour in that decreasing temperature caused a reduction in the expression peak of functional *HvLHY* whereas non-productive AS transcripts containing I4R, E6a and I8R increased at 4°C adding to the overall decrease in *LHY* mRNAs which code for the full-length LHY protein. We also showed that the E6a AS event in barley is NMD-sensitive as is also the case for E5a transcripts in orthologue *AtLHY* (James et al., 2012). Thus, *AtLHY* and *HvLHY* have similar gene expression and AS behaviour in response to lower temperature, even though specific AS events are not exactly conserved.

In contrast to *LHY*, low temperature induced different AS responses in *AtPRR7* and the barley orthologue *HvPPD-H1* (*HvPRR37*). *AtPRR7* had cold-dependent AS splicing events where exon 4 was skipped or intron 3 retained. Both unproductive transcripts greatly reduced the abundance of functional *AtPRR7* mRNA in the first day after transition to cold conditions [17]. These AS events are not conserved in the barley *HvPPD-H1* gene. Instead, *HvPPD-H1* had in-frame AS events that generated transcripts coding for different protein isoforms. At ambient temperatures *HvPPD-H1* potentially produces at least four different protein isoforms while at 4°C there was an AS isoform switch leading to a drastic reduction of specific isoforms coding for the longer forms of the protein. In addition, this switch was rapidly reversed (within 1 hour) when plants were returned to high temperatures. This illustrates the dynamic nature of AS and the potential for rapid responses at the AS level to changes in temperature. There are no known protein domains or functions in this region of the HvPPD-H1 protein, but the AS events were also observed in other monocot grass species suggesting that they may have a conserved function.

By directly comparing Arabidopsis clock AS events and behaviour, it was possible to identify conservation of some AS events at different levels using different definitions of AS conservation. For example, in identifying conservation of AS between Arabidopsis and *Brassica*, an AS event between homologues was considered to be conserved when the same type of event was present at the same splice-junction [43]. A more relaxed definition has been used by Chamala et al. [9] where the same type of AS event is present in the same region. Here, we found some AS events to be strongly conserved (the same event at the same site), and some where a similar event was found at the same region (e.g. *AtLHY* E5a and *HvLHY* E6a). However, we were also able to define conservation of AS behaviour and consequence even when AS events differed and were not conserved. For example, the cold-induced increase of non-functional *LHY* isoforms at the expense of functional mRNAs (e.g. *AtLHY* I1R and *HvLHY* I4R), even though the specific AS events were not conserved. It is also clear that some AS regulation in core clock genes was not conserved between barley and Arabidopsis. This probably indicates that AS represents an evolutionary strategy (in a similar way to gene duplication and diversification) that rapidly increases genome plasticity and develops new clock gene functions [37].

A number of the more abundant AS events at low temperature were intron retention events (e.g. I4R and I8R in *LHY*, and I1R in *PPD-H1*). Retention of intronic sequences is highly likely to introduce PTCs and in animals can trigger NMD. In plants, however the majority of intron retention transcripts are not affected by NMD [10,66]. It appears that such transcripts are retained in the nucleus and therefore avoid translation and the NMD machinery [73]. This may explain the high abundance of IR events detected in *HvLHY* (e.g. I4R) upon transition to cold and which were not sensitive to NMD in CHX treatment. The fate of these transcripts is unknown but an intriguing possibility is that under stress conditions, intron retention transcripts may be retained in the nucleus and when the stress is alleviated, intron removal is induced to give rapid production of functional mRNAs [74–76]. Such a hypothesis in circadian clock genes is interesting because the daily oscillations of protein expression levels require rapid and steep adjustments in mRNA levels [77].

The data presented here strongly suggest that AS provides an additional mechanism through which the barley clock is regulated when temperature changes. AS may fine-tune expression of genes in response to changing environmental cues and contributes to adaptation and speciation. Splicing and AS depends on sequence elements in the pre-mRNA and the interaction of trans-acting factors which bind to these cis-elements to define where the spliceosome assembles. The changes in clock AS may reflect features of the pre-mRNAs themselves (e.g. secondary structure) or temperature-dependent changes in levels and activity of trans-acting splicing factors or other factors [17]. Mutation and natural variation in gene sequences can affect splice site choice or efficiency of splicing thereby altering expression, which can affect fitness. The widespread nature of AS and the effect that subtle sequence changes can have in gene expression illustrates how AS is likely to have contributed significantly to plant adaptation [6]. Additionally, mutations in circadian clock genes have played a role in plant domestication and improvement of crop traits [21,78]. A future challenge will be to link genomic SNPs to variety- and species-specific AS, and to integrate transcription/AS data and protein isoform production to phenotypic variation. Quantitative RNA-seq at the transcript level combined with genome-wide association mapping may identify key functional AS variation [79,80]. The identification of clock gene regulation through AS in plants, combined with future experiments in natural conditions, may identify allele candidates/AS variants for plant improvement.

## Supporting Information

S1 Fig Sampling and temperature regime used in analyses of AS in regulating mRNA expression of clock genes. Sampling occurred 2.5 h after dawn. Red arrows represent time points when sampling occurred. Black boxes are for dark, white boxes are for light.
(PDF)

S2 Fig Nucleotide alignment of *HvPPD-H1* and *HvPP73* Exon 6 with homologous regions in *PRR3*, *PRR7*, *PRR37* and *PRR73* from eight other plant species. AS events were identified at the 5’ and 3’ ends of exon 6 in barley. **a** Alignments of the 5’ end of exon 6 with flanking intron sequences and **b** the 3’ end of exon 6 with flanking intron sequences are shown. Alignments were performed using the ClustalOmega software [63]. Exon sequence is represented by black upper-case letters, whereas introns are blue lower-case letters. The alternatively spliced regions described here for *HvPPD-H1* are highlighted in red in the *PRR37* orthologues and marked with arrows. Alternative splice site dinucleotides which are not conserved are highlighted in yellow. Plant species: At–*Arabidopsis thaliana*; Sl–*Solanum lycopersicum*; St–*Solanum tuberosum*; Sb–*Sorghum bicolor*; Zm–*Zea mays*; Os–*Oryza sativa*; Bd–*Brachypodium distachyon*; Hv–*Hordeum vulgare*; Ta–*Triticum aestivum*.
(PDF)

S3 Fig Genomic structure and conserved AS events of Arabidopsis and barley *GI* and *TOC1*, and *HvCO2*. In the Arabidopsis genes, only those AS events which show conservation to barley are shown—for other Arabidopsis clock genes AS events see James et al. (2012a). Other abundant AS events in barley are also shown. Exons are numbered; 5’ and 3’ UTRs are dark boxes; coding sequences are open boxes. Alt, alternative; ss, splice site; I, intron; R, retention; ES, exon skipping; AUG, translation start site. Small black lines on the top right of each gene structures represent a scale of 200 bases.
(PDF)

S4 Fig Confirmation of NMD-impairment in the cycloheximide (CHX) assay by HR RT-PCR of *HvELF3* transcripts. CHX treatment performed at both 20 and 4°C conditions. The barley line homozygous for the PTC-containing allele *Hvelf3* (blue) showed increased abundance on CHX treatment (* represents *P* < 0.001) suggesting NMD is impaired upon CHX treatment. WT and Bowman (*ppd-H1*) have the wild-type (non-mutant) *HvELF3* allele and, as expected, does not show increased *HvELF3* abundance on CHX treatment.
(PDF)

S5 Fig Cold-dependent *LHY* AS E6a transcripts detected by RT-PCR using primers located in E6 (forward) and E6a (reverse). Samples were harvested in the morning (2.5 h after dawn) in six different time-points/temperatures: 20°C (Day 7), Day 1 at 4°C, Day 2 at 4°C, 4°C (Day 4), Day 1 at 20°C and 20°C (Day 2). Amplification of UBC (*HvUBC21*) and PP2A (*HvPP2AA2*) served as reference genes. Three biological replicates were analysed. 5’ and 3’ UTRs are open boxes; coding sequences are dark boxes, except E6a, which is shaded yellow. 812, unspliced product; 640, E6a-containing product; -, negative control (RNA template).
(PDF)

S6 Fig Expression changes of clock genes during transfer from 20°C to 4°C. Total transcript levels of **a** *HvLHY*, **b** *HvPPD-H1* and **c** *HvPRR73* in the morning (2.5 h after dawn) at six different time-points/temperatures: 20°C (Day 7), Day 1 at 4°C, Day 2 at 4°C, 4°C (Day 4), Day 1 at 20°C and 20°C (Day 2). Results obtained with different HR RT-PCR primer pairs were considered as technical replicates. Error bars: SEM from three biological replicates. Comparisons are of clock gene levels between *Hvelf3*, WT and Bowman (*ppdh1*). *, *P* < 0.05; **, *P* ≤ 0.005; ***, *P* < 0.001.
(PDF)

S7 Fig HR RT-PCR analysis of AS in the MYB-coding domain region of *HvLHY*. Exons are numbered on the genomic structure; 5’ UTR is the dark box; coding sequences are open boxes. Diagonal lines represent splicing events. AS events are shown in red. Approximate positions of primers are shown by arrowheads. Representative electropherograms of spectral data collected during the sample run and produced by GeneMapper^®^ show the size of detected peaks corresponding to RT-PCR products from *LHY* fully spliced (FS) and AS transcripts (alternative 3’ splice site in exon 4 –Alt3’ss E4 and intron 4 retention—I4R).
(PDF)

S1 File Report on some of the low abundance unproductive AS events in barley clock genes. (DOCX)

S1 Table Barley gene-specific primers and their sequences used for expression and AS analyses. (PDF)

S2 Table Barley clock gene-specific primers used in AS analyses and their sequences. (PDF)

S3 Table AS events in barley clock and clock-related genes detected by HR RT-PCR experiments. (PDF)
