# Supplementary figures and images for: Alternative Splicing of Barley Clock Genes in Response to Low Temperature
Source: PLoS One. 2016 Dec 13;11(12):e0168028. doi: 10.1371/journal.pone.0168028 (PMC5154542; doi:10.1371/journal.pone.0168028)

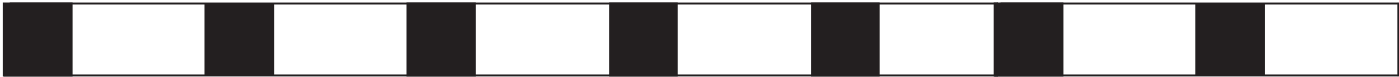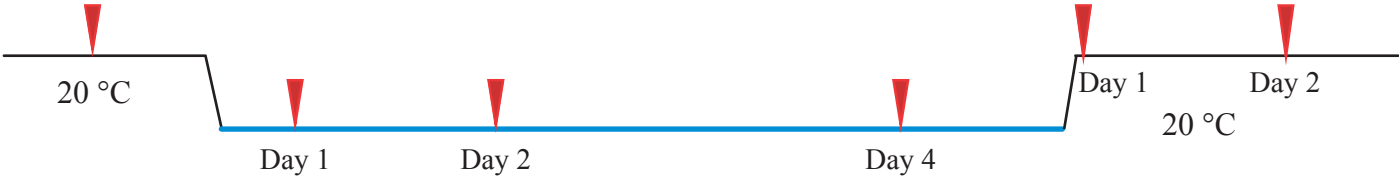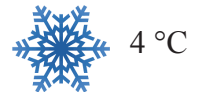

Supplement: S1 Fig — Sampling occurred 2.5 h after dawn. Red arrows represent time points when sampling occurred. Black boxes are for dark, white boxes are for light. (PDF) [file pone.0168028.s001.pdf]

*AtGI*

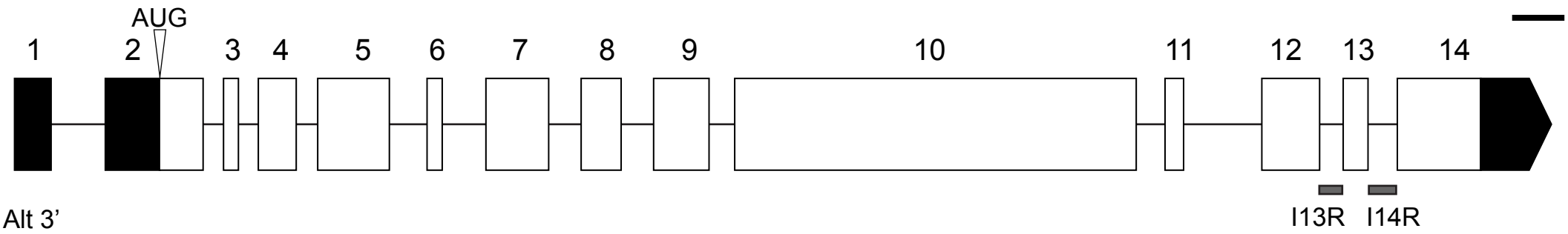

*HvGI*

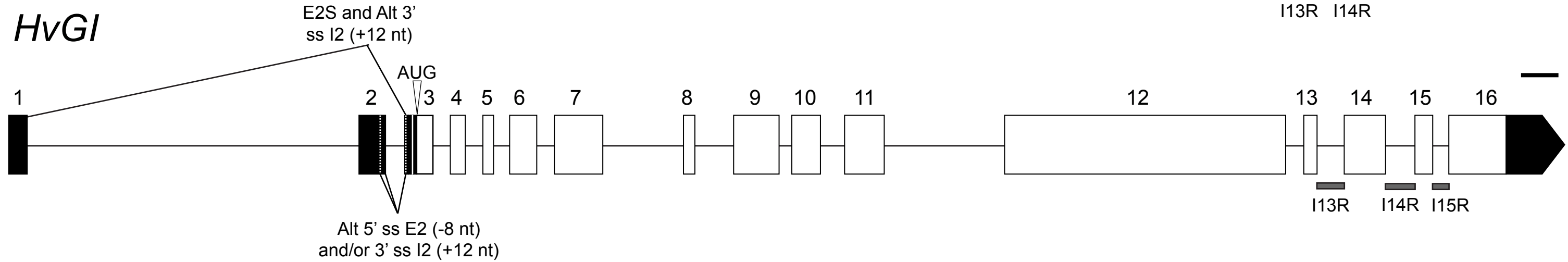

*HvCO2*

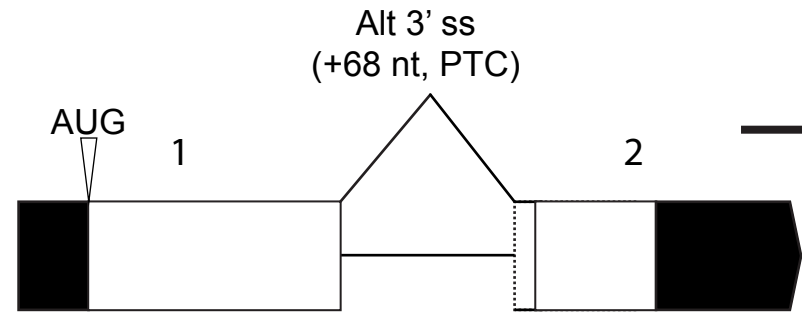

*AtTOC1*

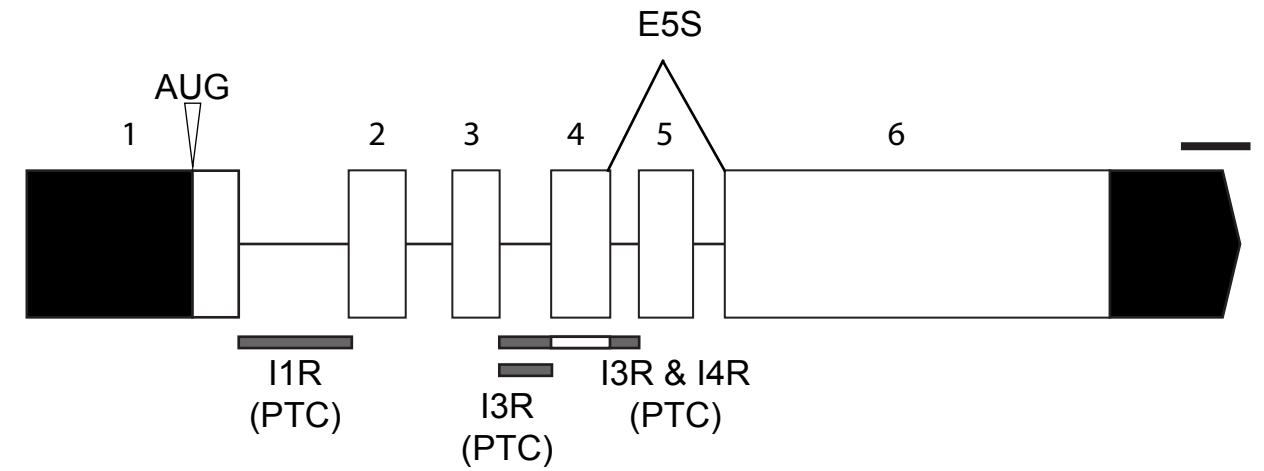

*HvTOC1*

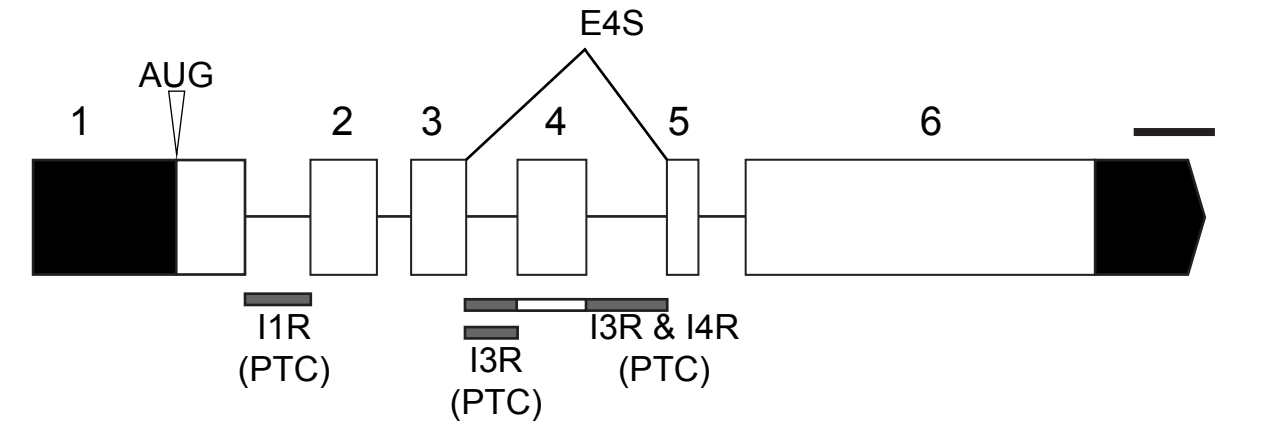

Supplement: S3 Fig — In the Arabidopsis genes, only those AS events which show conservation to barley are shown—for other Arabidopsis clock genes AS events see James et al. (2012a). Other abundant AS events in barley are also shown. Exons are numbered; 5’ and 3’ UTRs are dark boxes; coding sequences are open boxes. Alt, alternative; ss, splice site; I, intron; R, retention; ES, exon skipping; AUG, translation start site. Small black lines on the top right of each gene structures represent a scale of 200 bases. (PDF) [file pone.0168028.s003.pdf]

***ELF3* relative expression**

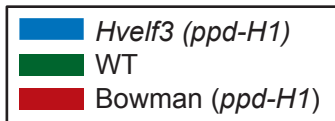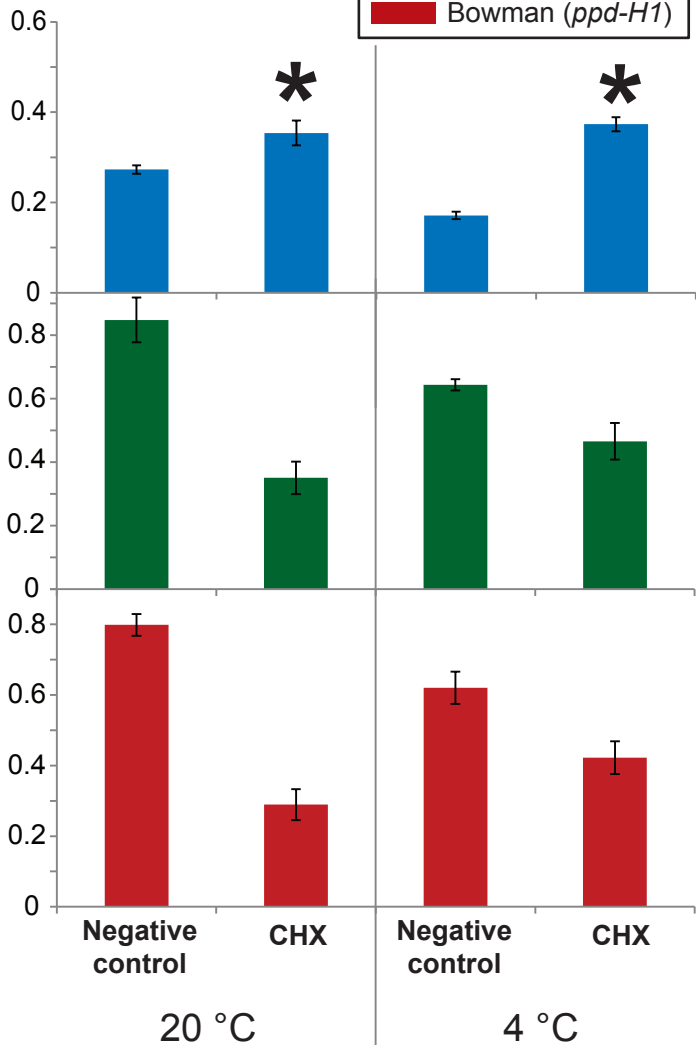

Supplement: S4 Fig — CHX treatment performed at both 20 and 4°C conditions. The barley line homozygous for the PTC-containing allele Hvelf3 (blue) showed increased abundance on CHX treatment (* represents P < 0.001) suggesting NMD is impaired upon CHX treatment. WT and Bowman (ppd-H1) have the wild-type (non-mutant) HvELF3 allele and, as expected, does not show increased HvELF3 abundance on CHX treatment. (PDF) [file pone.0168028.s004.pdf]

HvLHY

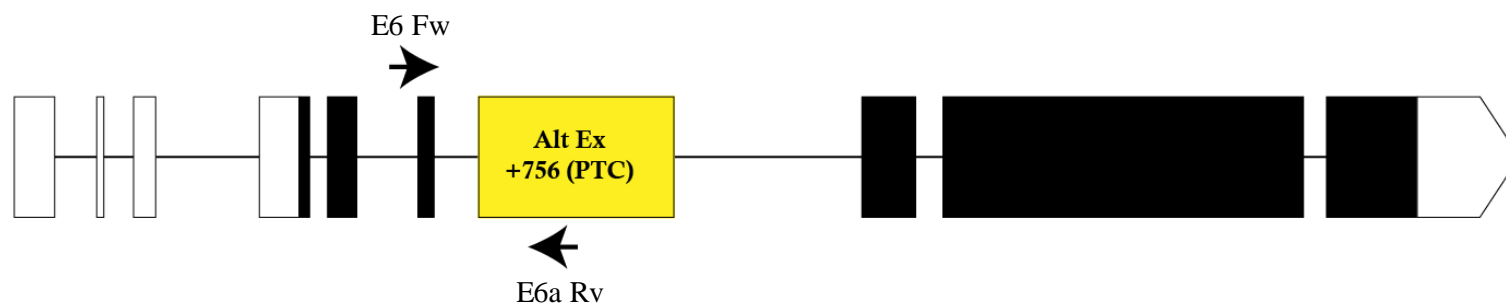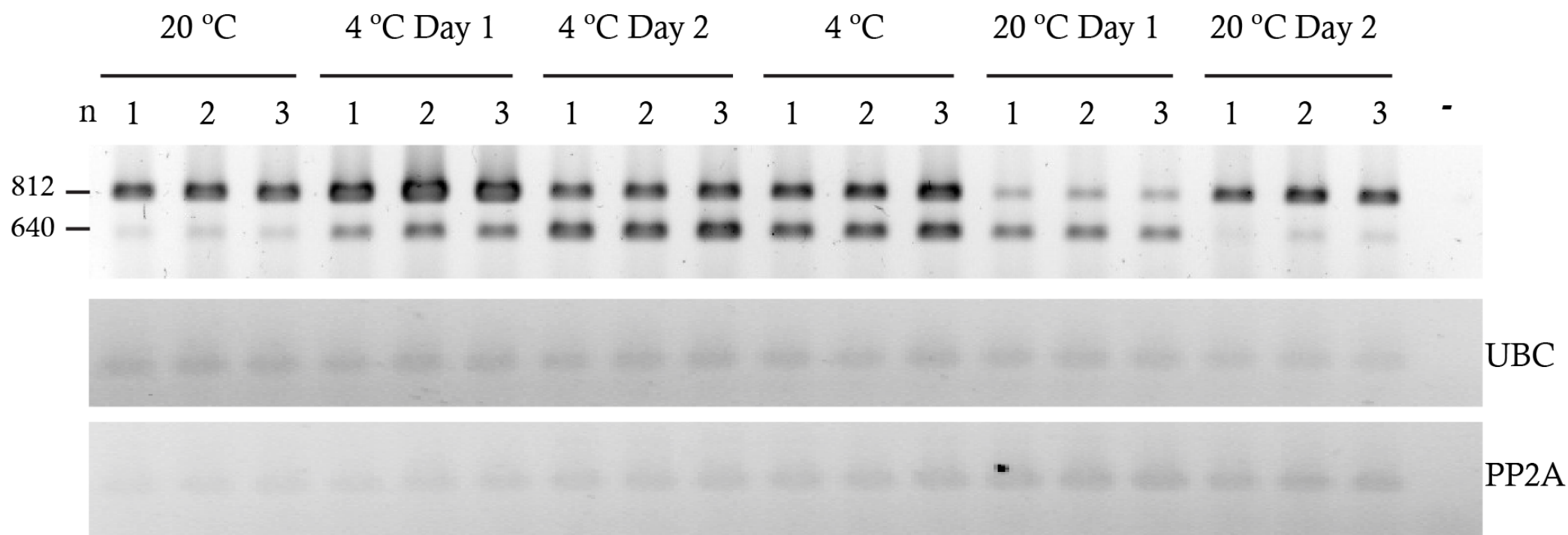

Supplement: S5 Fig — Samples were harvested in the morning (2.5 h after dawn) in six different time-points/temperatures: 20°C (Day 7), Day 1 at 4°C, Day 2 at 4°C, 4°C (Day 4), Day 1 at 20°C and 20°C (Day 2). Amplification of UBC (HvUBC21) and PP2A (HvPP2AA2) served as reference genes. Three biological replicates were analysed. 5’ and 3’ UTRs are open boxes; coding sequences are dark boxes, except E6a, which is shaded yellow. 812, unspliced product; 640, E6a-containing product; -, negative control (RNA template). (PDF) [file pone.0168028.s005.pdf]

**a***HvLHY* total transcripts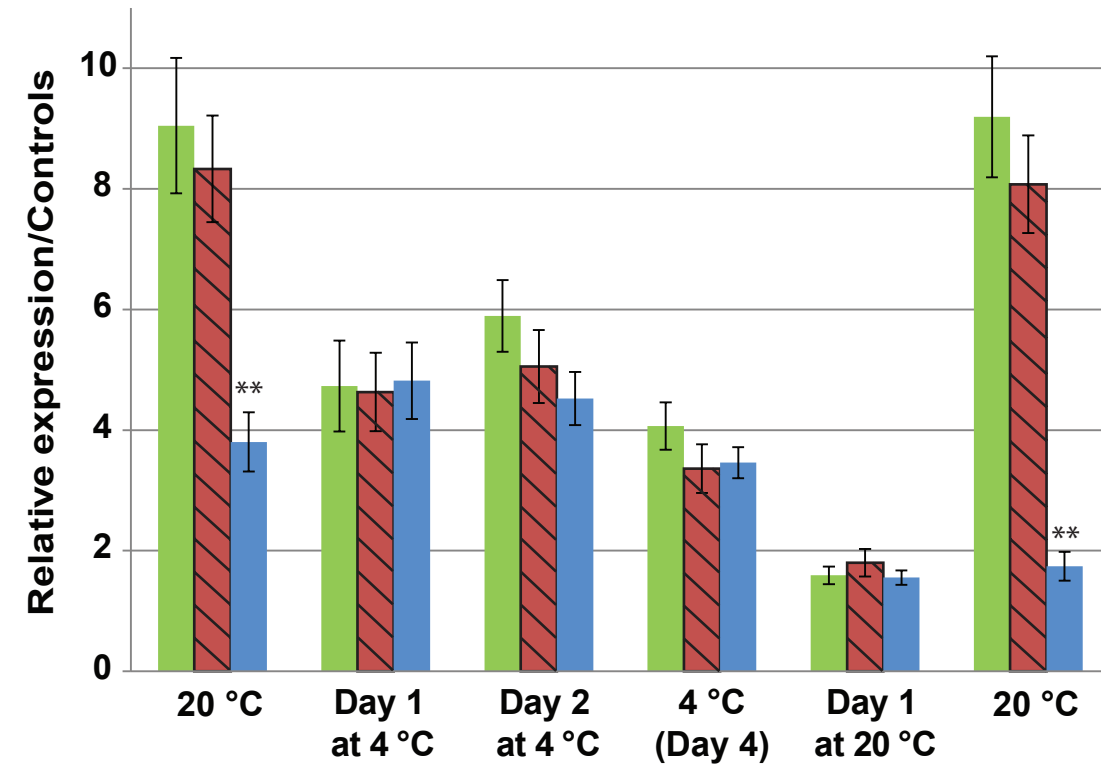**b***HvPPD-H1* total transcripts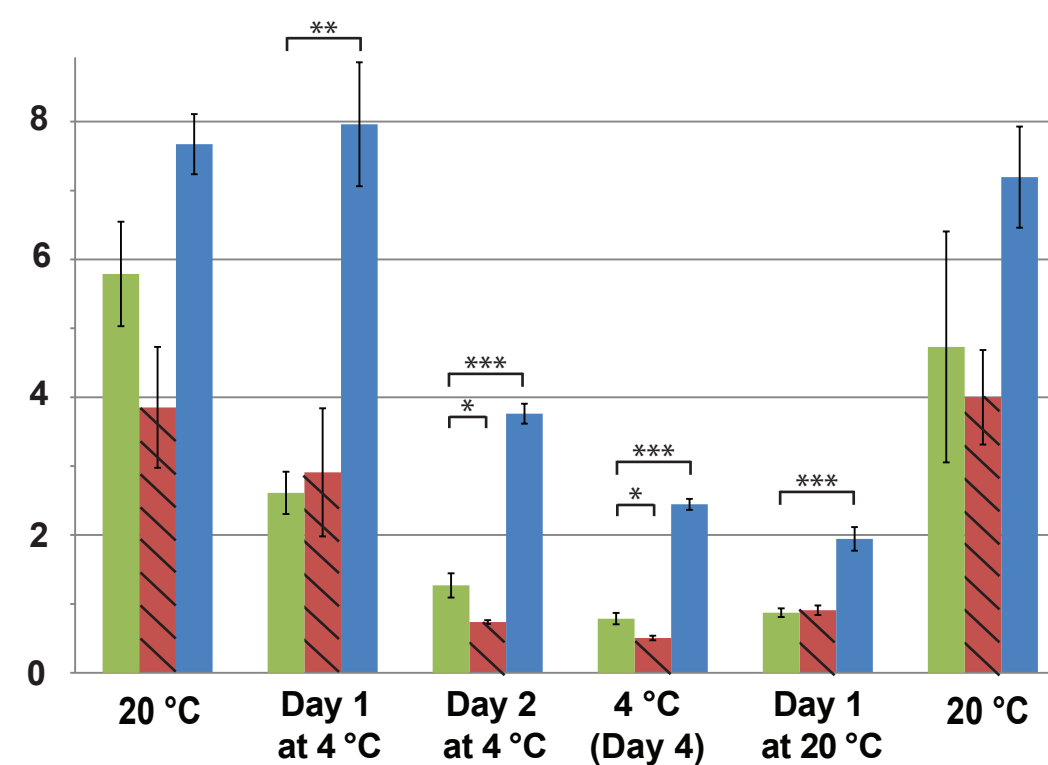**c***HvPRR73* total transcripts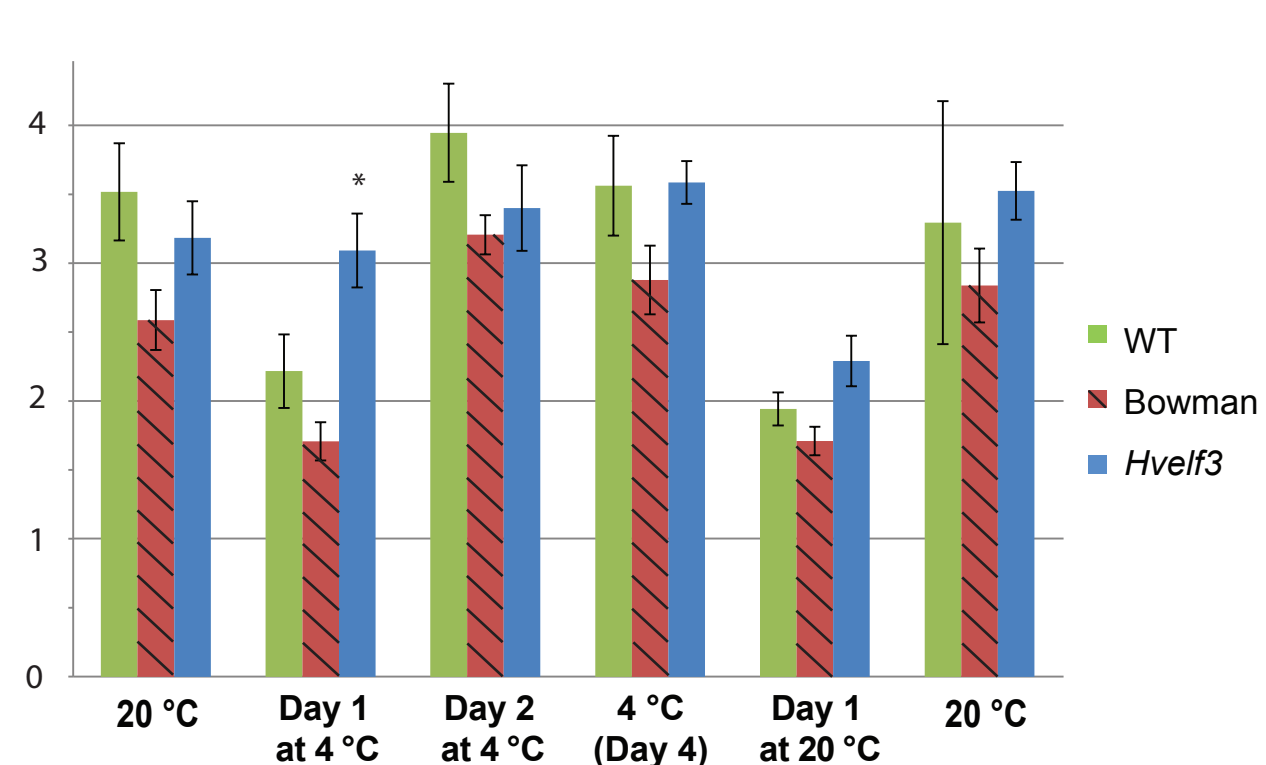

Supplement: S6 Fig — Total transcript levels of a HvLHY, b HvPPD-H1 and c HvPRR73 in the morning (2.5 h after dawn) at six different time-points/temperatures: 20°C (Day 7), Day 1 at 4°C, Day 2 at 4°C, 4°C (Day 4), Day 1 at 20°C and 20°C (Day 2). Results obtained with different HR RT-PCR primer pairs were considered as technical replicates. Error bars: SEM from three biological replicates. Comparisons are of clock gene levels between Hvelf3, WT and Bowman (ppdh1). *, P < 0.05; **, P ≤ 0.005; ***, P < 0.001. (PDF) [file pone.0168028.s006.pdf]

# *HvLHY* Exon 4 - Exon 6

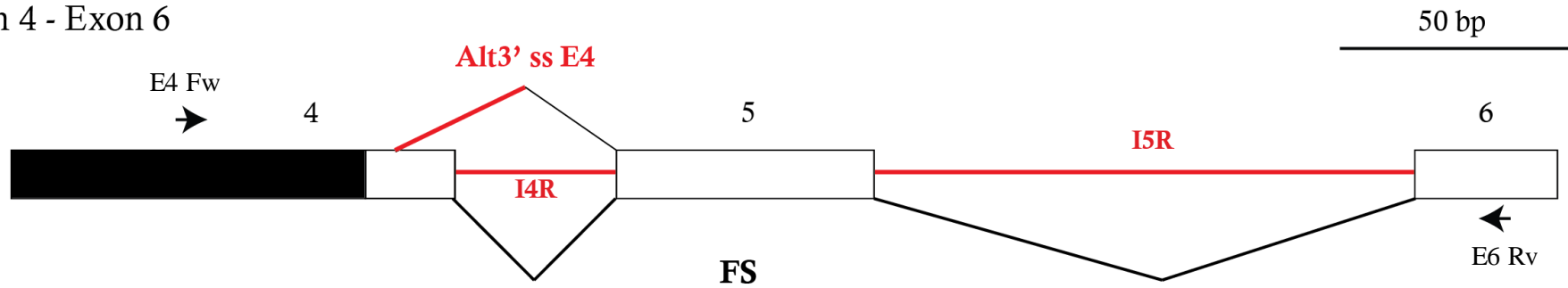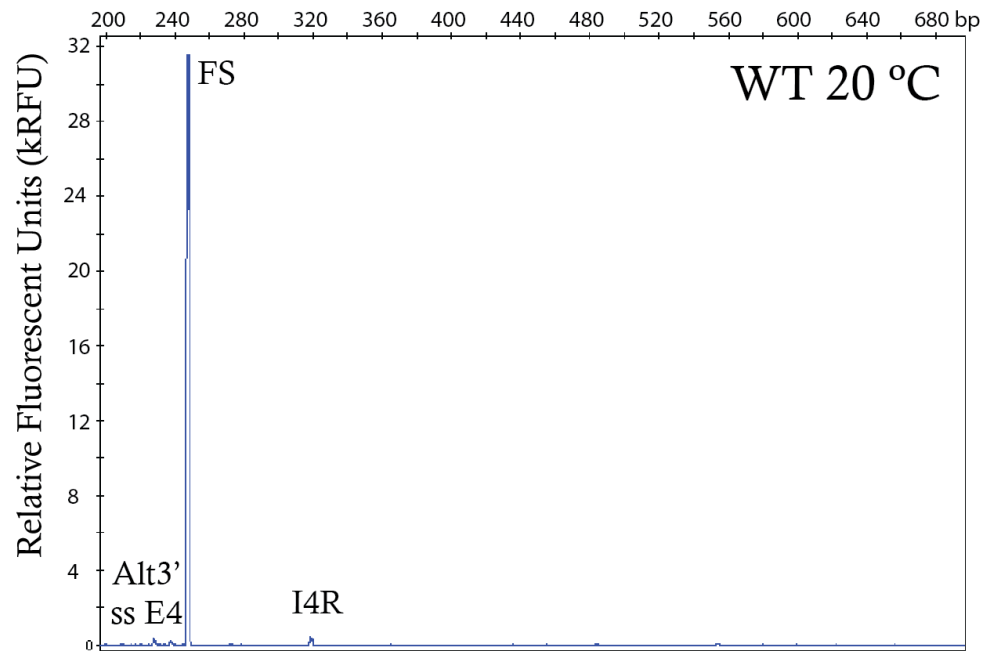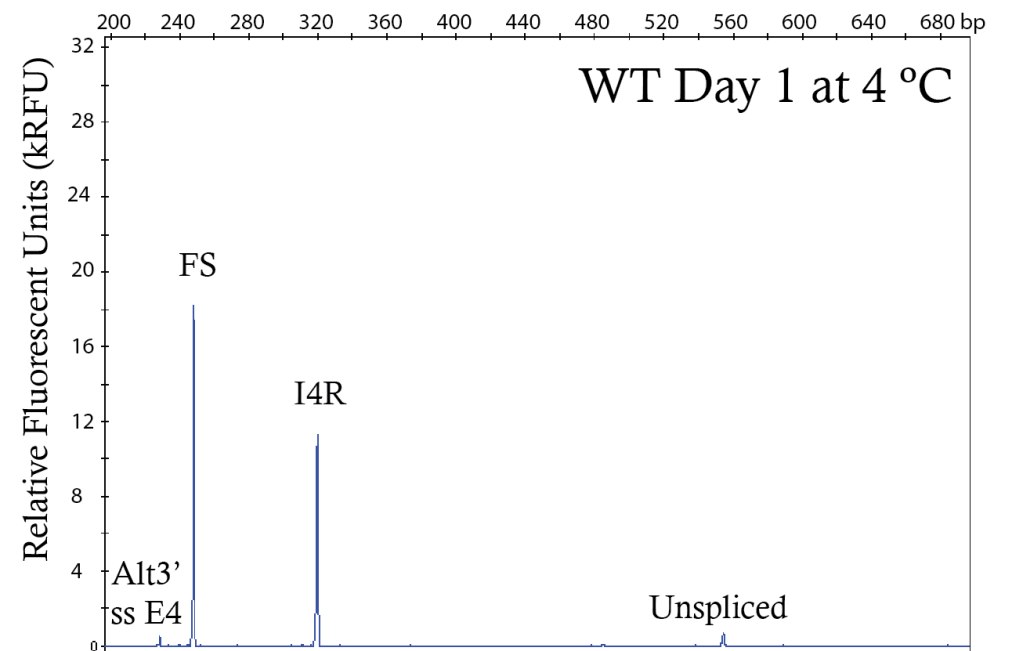

1  
2  
3

Supplement: S7 Fig — Exons are numbered on the genomic structure; 5’ UTR is the dark box; coding sequences are open boxes. Diagonal lines represent splicing events. AS events are shown in red. Approximate positions of primers are shown by arrowheads. Representative electropherograms of spectral data collected during the sample run and produced by GeneMapper® show the size of detected peaks corresponding to RT-PCR products from LHY fully spliced (FS) and AS transcripts (alternative 3’ splice site in exon 4 –Alt3’ss E4 and intron 4 retention—I4R). (PDF) [file pone.0168028.s007.pdf]
