# Supplementary material for: Alternative Splicing of Barley Clock Genes in Response to Low Temperature
Source: PLoS One. 2016 Dec 13;11(12):e0168028. doi: 10.1371/journal.pone.0168028 (PMC5154542; doi:10.1371/journal.pone.0168028)
Supplement: S2 Fig — AS events were identified at the 5’ and 3’ ends of exon 6 in barley. a Alignments of the 5’ end of exon 6 with flanking intron sequences and b the 3’ end of exon 6 with flanking intron sequences are shown. Alignments were performed using the ClustalOmega software [63]. Exon sequence is represented by black upper-case letters, whereas introns are blue lower-case letters. The alternatively spliced regions described here for HvPPD-H1 are highlighted in red in the PRR37 orthologues and marked with arrows. Alternative splice site dinucleotides which are not conserved are highlighted in yellow. Plant species: At–Arabidopsis thaliana; Sl–Solanum lycopersicum; St–Solanum tuberosum; Sb–Sorghum bicolor; Zm–Zea mays; Os–Oryza sativa; Bd–Brachypodium distachyon; Hv–Hordeum vulgare; Ta–Triticum aestivum. (PDF) [file pone.0168028.s002.pdf]

a

```
AtPRR3    ttgattggttttgcatttataaaaatatgtagAGTTCTTGGACAAAAAGAG
AtPRR7    aggttgattttgacatacctttttttgttcagAGCTCTTGGACGAAAAAAG
SlPRR7    tcttttctgagtctggaatttgtattttgcagAATTCCTGGACAAAGCAAG
StPRR7    ttctttctgagtctgaaatttgtattttgcagAATTCCTGGACAAAGCAAG
SlPRR3    tataaatcatcttcttatttctttttggcagAGTTCGTGGTCTAAAAGGG
StPRR3    tataaatcatcttcttatttctttttggcagAGTTCATGGTCTAAAAGGG
SbPRR73   ttatcgagtgcacaatctgttttaaaatgcagAGCTCATGGACAAAACGTG
ZmPRR73   ttatcgagtgcacaatctgtttcaaaatgcagAGCTCATGGACAAAGCGTG
OsPRR73   ttctcatgccatcagcctgttttaaaacacagAGTTCATGGACAAAGCGTG
BdPRR73   cgatatatatgtatatatattttcaaatgcagAGTTCGTGGACAAAGCGTG
HvPRR73   ttctcatgccactgatctattttaaaatgcagAGTTCATGGACGAAGCGTG
AtPRR73   --------------------------------AGTTCATGGACGAAGCGTG
HvPPDH1   ctagtttctcatcaatttattttaagGCGCAGAGCTCATGGACAAAGCGTG
TaPpd-A1  tagttttctcatcaatttattttaaggcgcagAGCTCATGGACAAAGCGTG
TaPpd-D1  cagttttctcatcaacttattttaaggcgcagAGCTCATGGACAAAGCGTG
TaPpd-B1  aagttttctcatcaatttattttaacgcgcagAGCTCATGGACAAAGCGTG
BdPRR37   ctgttttctcatcagtctattttaactcacagAGCTCATGGACAAAGCTTG
OsPRR37   aactggttgctcatcatttttttaagGCGCAGAGCTCATGGACAAAGCGCG
SbPRR37   ttgttccctcattaatctgttacaagGCGCAGAGCTCATGGACTAAGTGTG
ZmPRR37   ttgtttactggttaatatgtcataagGCACAGAACTCGTGGACTAAGCTTG
```

3’ss　alt 3’ss

b

```
AtPRR3    AGGAACAAATAGgtaagaacgcaaattaatta-----gtcaattaaaatgtgagtacactgatcttgtggaacaat
AtPRR7    AGTAATCAGTTGGTTGCACCACCTGCTGAGAAGGAGACTCAAGAACATGATGATAA---ATTTGgtaaagaaagga
SlPRR7    GCTTACAAGAATGCACAGAGAAATGCCAAAAGGAAATGCCAAGAAGAAGAAGAACATCCTGgtaatgagtatttct
StPRR7    GCTAACAAGAATTCACAGAGAAATGCCAAAAGGAAATGCCAAGAAGAAGAACATCCTGgtaatgagtatttctaat
SlPRR3    AGTGCTACCTGGGTGCCTGCAAATGCCACAAGAAGATGTCTCAATGAGAAGGATGAACTGGgtaggtggctcttcc
StPRR3    AGTGCTACCTGGGTGCCTGCAAATGCCACCAGAAAATGCCTCAATGAGAAGGATGAACTGGgtaggtggctcttcc
SbPRR73   AGTAACAAGTGGCTACCGACAGCAAACAAAAGGAATGGCAAGAAACATAAGGAGAA---TAAAGgtatcatatgct
ZmPRR73   AGTAACAAGTGGTTACCGACAGCAAACAAAAGGAATGTCAAGAAACAGAAGGAGAA---TAAAGgtatatgctcca
OsPRR73   AGCAACAGGTGGTTACCGACTGCAAATAAAAGGAGCGGAAAGAAACATAAAGAAAA---TAACGgtactatgcttg
BdPRR73   AGCAATAGGTGGTTGCCAACTGAAAATAAAAGAAACAGTAATAATCAAAAAGAGAG---TAATGgtatgctcgata
HvPRR73   AGCAATAGGTGGTTGCCGACTGCAAATAAAAGGAATATCAATAATCAAAAAGAAAA---TAATGgtatcgtattct
AtPRR73   AGCAATAGGTGGTTGCCGACTGCAAATAAAAGGAACATCAATAATCAAAAAGAAAA---TAATG------------
HvPPDH1   AGCAATAGATGGTTACCAGGTACAAATAACAAAAAATGCCAGAAACCAAAAGAAACCACTAATGgtatggtatgct
TaPpd-A1  AGCAACAGATTAA---GAGGTACAGATAACAAAAAATGCCAGAAACCAAAAGAAAC---TAATGgtatggtatgct
TaPpd-D1  AGCAATAGATTAA---GAGGTACAAATAACAAAAAATGCCAGAAACCAAAAGAAAC---TAATGgtatggtatgct
TaPpd-B1  AGCAATAGATTAA---GAGGTACAGATAACAAAAAATGCCAGAAACCAAAAGAAAC---TAATGgtatggtatgct
BdPRR37   AGCAATAGATGGTTACCAGGTACAAACAACAAAAAATGCAAGAAACAAAAGGAAAA---TAATGgtatggtatgct
OsPRR37   AGCAATAGATGGTTACCATGTACAAGCAACAAAAATTCCAAGAAACAAAAAGAAAC---TAATGgtattgtatgct
SbPRR37   AGCAACAGACGGCTACCAGGTACGAGCAACAGAAACTGCATGAAGCAAAAATACAC---TAATGgtactgattcac
ZmPRR37   AGCAACAGATGGCTACCAGATAAGAGCAACAGAAACTGCAAGAAGCCAAAAAACAC---TAATGgtatgtcatgct
```

alt 5’ss　5’ss
