# Supplementary material for: Alternative Splicing of Barley Clock Genes in Response to Low Temperature
Source: PLoS One. 2016 Dec 13;11(12):e0168028. doi: 10.1371/journal.pone.0168028 (PMC5154542; doi:10.1371/journal.pone.0168028)
Supplement: S1 File — (DOCX) [file pone.0168028.s008.docx]

**S1 File**

Journal: *PLoS ONE*

Title: **Alternative splicing of barley clock genes in response to low temperature**

Authors: Cristiane P. G. Calixto^1^, Craig G. Simpson^2^, R. Waugh^1,2^, and John W. S. Brown^1,2^

^1^Division of Plant Sciences, School of Life Sciences, University of Dundee at the James Hutton Institute, Invergowrie, Dundee DD2 5DA, Scotland.

^2^Cell and Molecular Sciences, The James Hutton Institute, Invergowrie, Dundee DD2 5DA, Scotland.

Corresponding author: Prof. J.W.S. Brown

e-mail: [j.w.s.brown@dundee.ac.uk](mailto:j.w.s.brown@dundee.ac.uk); [John.Brown@hutton.ac.uk](mailto:John.Brown@hutton.ac.uk)

**Supporting Results**

The analysis of AS in barley clock genes identified 59 alternative splicing events in addition to the 12 fully spliced transcripts from eleven clock genes (Table 1). Of the AS events, twelve were found at levels of between 11-76% of the total transcripts in WT plants grown at 20 °C or 4 °C. These abundant transcripts were found in four different genes: *HvLHY*, *HvPRR37*, *HvGI* and *HvCO2* and are described in the main text (Table 1). The remaining lower abundance AS events in these and the other clock genes are described below.

***HvLHY***

Ten AS events were observed for barley *LHY* of which six were within the complex 5’ UTR (four exons and three introns in the current annotation) (Fig 1; S3 Table). Five of the 5’UTR events involved retention of either intron 1, 2 or 3 or combinations of these. The sixth 5’UTR event is an alternative 3’ splice site (3’ss) in intron 3 which adds 80nt. The six AS events in the 5’ UTR introduce different numbers of upstream open reading frames (uORFs) (Table 1) of up to 31 amino acids.

***HvPRR37 and HvPRR73***

The barley paralogues *PRR37* and *PRR73* undergo nine and seven AS events, respectively (S3 Table). In the 5’ UTR of *PRR37*, a cryptic intron (CrIn) in Exon 1 was observed in about 20% of the reads from the RNA-seq data [1], while the other 80% reads retained the CrIn (Fig 1). Removal of the CrIn resulted in the loss of a uORF coding for 48 amino acids, which may affect translation of the functional ORF. Two low-abundant AS events in Exon 6 of PPD-H1 were identified: Alt 5’ ss adding 5 nt to Exon 6 and I6R. Both AS events caused the introduction of PTCs such that they do not code for full-length PRR37 protein. These PTC-containing transcripts represent around 7.7% of total transcripts at 20 °C and become undetectable upon transfer and acclimation to 4 °C. Barley plants treated with CHX did not have significantly higher levels of I6R or alt 5’ss I6 (data not shown), suggesting they are not targeted to NMD degradation. Five of the seven AS events in *HvPRR73* were low abundance IR events in the coding region which may represent partially spliced transcripts.

We also analysed dynamic changes on expression and AS *HvPRR73* under low temperature. *PRR73* mRNA levels in WT plants decreased from 100% at 20 °C down to around 65% on Day 1 at 4 °C. Upon cold acclimation, the transcript levels returned to those at 20 °C suggesting that *PRR73* expression was affected during temperature transitions rather than temperature acclimation (Fig 3c). Analysis of AS by HR RT-PCR, using primers spanning most of the *PRR73* gene sequence did not detect any highly significant cold-dependent AS event. Therefore, the paralogues *PPD-H1* and *PRR73* behaved very differently in their AS response to low temperature.

***HvCO2***

Three AS events were observed using *HvCO2*-specific primers: 1) IR1, 2) alt 3’ ss and 3) cryptic intron (CrIn) removal (Table 1, S3 Fig, S6 Table). However, the unspliced (IR) and CrIn transcript isoforms were generated from antisense transcripts from a gene on the opposite strand as has also been seen for *AtCOL1*. For example, the CrIn contained canonical splice sites (GT-AG) in the complementary orientation and expression of IR and CrIn transcripts were rhythmically opposite to the spliced and alt 3’ ss *HvCO2* transcripts in light/dark conditions (unpublished data). These transcripts were not considered further.

***HvTOC1/PRR1***

*HvTOC1* has three intron retention and one exon skipping event (S2 Fig, S5 Table). The I1R, I3R and I3R+I4R events disrupt the coding region of mRNAs from both *AtTOC1* and *HvTOC1* orthologues and the exon skipping (ES) event was identified for exons 5 and 4 in Arabidopsis and barley *TOC1*, respectively. Skipping of exon 4 in *HvTOC1* removes 168 nt in frame. Both IR and ES events are of low abundance in barley and Arabidopsis plants grown at 20 °C but levels of *AtTOC1* transcripts retaining I4 transiently increase during cold acclimation [2].

***HvPRR59 and HvPRR95***

Although AS events of *HvPRR59* and *HvPRR95* were not abundant, many were similar to AS events described in *PRR5* or *PRR9* of Arabidopsis [2]. Both barley homologues, *PRR59* and *PRR95*, undergo five AS events (S6 Table). The C-terminal coding regions in *AtPRR9* and both barley homologues have conserved IR events that would be unable to code for the CT domain. Interestingly, I2R (*HvPRR95*) and I3R (*HvPRR59*) events are similar to the cold-dependent I3R AS events in *PRR9* [2], which is also affected in *prmt5* and *stipl1* mutants [3,4].

***HvELF3 and HvGRPs***

AS analyses of the *HvELF3* gene identified retention of intron 3, which disrupts the coding region by introducing PTCs. Single intron genes *HvGRP7a* and *HvGRP7b* also undergo IR events, according to RNA-seq data (data not shown).

**Supporting References**

1. Mayer KF, Waugh R, Brown JW, Schulman A, Langridge P, Platzer M, et al. A physical, genetic and functional sequence assembly of the barley genome. Nature. 2012;491: 711-716.

2. James AB, Syed NH, Bordage S, Marshall J, Nimmo GA, Jenkins GI, et al. Alternative splicing mediates responses of the Arabidopsis circadian clock to temperature changes. Plant Cell. 2012;24: 961-981.

3. Sanchez SE, Petrillo E, Beckwith EJ, Zhang X, Rugnone ML, Hernando CE, et al. A methyl transferase links the circadian clock to the regulation of alternative splicing. Nature. 2010;468: 112-116.

4. Jones MA, Williams BA, McNicol J, Simpson CG, Brown JWS, Harmer SL. Mutation of Arabidopsis spliceosomal timekeeper locus1 causes circadian clock defects. Plant Cell. 2012;24: 4066-4082.
