# Supplementary material for: Alternative Splicing of Barley Clock Genes in Response to Low Temperature
Source: PLoS One. 2016 Dec 13;11(12):e0168028. doi: 10.1371/journal.pone.0168028 (PMC5154542; doi:10.1371/journal.pone.0168028)
Supplement: S1 Table — (PDF) [file pone.0168028.s009.pdf]

Table S1. Barley gene-specific primers and their sequences used for expression and AS analyses.

Genes	Primer name	Sequence (5' → 3')
LHY	HvLHY-Ex6Fw	AACAAAGCGACCAGTTGTCAGCAC
	HvLHY-Ex8Rv	TGAGCATGGCTTCTGATTTGCACG
TOC1	HvPRR1-Ex3Fw	TCTGTTGTTGTCAAGTGCTTGCGG
	HvPRR1-Ex6Rv	CAATGAGCTGTTTGCTGGAGTGCT
PPD-H1	HvPpdH1-Ex1Fw	GGAGTTCCTACACAGGAAGACCAT
	HvPpdH1-Ex4Rv	TCGTCGTTGCGATCATTGCTGTTG
PRR59	HvPRR59-Ex7Fw	TGGACTGTGAGCGGTGAAACTGAT
	HvPRR59-3UTRv	TCACCTGGGAAGGGAGGTATCAAA
PRR95	HvPRR95-Ex6FwB	GCTGAACAACTCTGGCAACAGCAA
	HvPRR95-3UTRv	TTCAAAGGACACACCAAATCGGCG
PRR73	HvPRR73-Ex3Fw	TTAACTGAGGTCGCCATGCCTTGT
	HvPRR73-Ex5Rv	TCATCATGACTGCCGCCACTGTTT
LUX	HvLUXFw	GGCTCCTTCCCGTCCTTCC
	HvLUXRv	TTGACGTAGAGGCGGTACTTCTGGA
ELF3	HvELF3-Ex2Fw	TCTCCAGATGATGTTGTCGGTGCT
	HvELF3-Ex4Rv	TGAGATGGTGAAGCCTGGTTTCCT
ZTLa	HvZTLa-5UTRFw	AGGAGGAGGGATAGGAGGCG
	HvZTLa-Ex2Rv	AGGGGTCAGATGCAACCTGT
FKF1	HvFKF1-Ex1Fw	CGGGGCTGGGGATGGCAGGC
	HvFKF1-Ex2Rv	ATCGGTTGTTTACTGGAGCT
GI	HvGI-Ex14Fw	TCGTGCTACAGATGGGATGCTTGT
	HvGI-Ex16Rv	GTCCACTGTTCAAGATGTCACGGA
CABa	HvCABa-5UTRFw	AACACACGACACACCTCTAGCCTT
	HvCABa-3UTRv	TGTGCAGTTCACTGTAGTCGCCTT
CO1	HvCO1-Ex1Fw	TGTGAGAAGGCCACATCAGAGTGG
	HvCO1-Ex2Rv	ATGTCTGGTCCACTTCCATTTCCGC
CO2	HvCO2-Ex1FwB	GAGCAGCAAGGGAGCAATTA
	HvCO2-3UTRv	TACGGTGCCCATCATTCCATGTGT
ELF4-likeA	HvELF4likeAFw	ATATCCATCTTCCTCCCGCACGCA
	HvELF4likeARv	ATGGTCTGCCTCCTCTACTGTGT
FT1	HvFT1-5UTRFw	ACAGCTTACATTGCTGCTCTCTGC
	HvFT1-3UTRv	TGGATAATTTGGTGACTTGGCGGC
PP2AA2	HvPP2AA2-Ex2Fw	CCGCATTGGTGCACAGATGAAAGA
	HvPP2AA2-Ex3Rv	CCAAGATTTGATGCAGCTGCCCTT
UBC21	HvUBC-Ex3Fw	AGTACAAGGAGGTGCAGCGAGAAA
	HvUBC-Ex4Rv	CTGCTCGGGAATTGAGAATGCAAG

Primer name includes directions: Fw is Forward, whereas Rv is Reverse.
