# Supplementary material for: Alternative Splicing of Barley Clock Genes in Response to Low Temperature
Source: PLoS One. 2016 Dec 13;11(12):e0168028. doi: 10.1371/journal.pone.0168028 (PMC5154542; doi:10.1371/journal.pone.0168028)
Supplement: S2 Table — (PDF) [file pone.0168028.s010.pdf]

Table S2. Barley clock gene-specific primers used in AS analyses and their sequences.

Genes	Primer name	Sequence (5' → 3')
LHY	HvLHY-Ex3Fw	GCAAAGTACTGGAATTTCTGAGCCC
	HvLHY-Ex8Fw	CGTGCAAATCAGAAGCCATGCTCA
	LHY-Ex10FwC	GCTAACGACAAGACACAGCAAGCA
	HvLHY-Ex6Rv	GTGCTGACAACTGGTCGCTTTGTT
	LHY-Ex9Rv	TTGGAACTTCTCTGGTGGGTGTCT
	HvLHY-Ex8aRv	AGGACAAGCAGCAAAGAACAGAA
	LHY-3UTRv	GGGCTCAGAAATTCCAGTACTTTGC
TOC1	HvPRR1-5UTRFwB	GCCGGACTGCGACGGGCGG
	HvPRR1-Ex1Fw	ATGTGCTTCGCCTCCTCTGTAACT
	HvPRR1-Ex6Fw	CAAGCACTCCAGCAAACAGCTCAT
	HvPRR1-Ex1Rv	AGTTACAGAGGAGGCGAAGCACAT
	HvPRR1-Ex3Rv	CCGCAAGCACTTGACAACAACAGA
	HvPRR1-Ex6Rv	CAATGAGCTGTTTGCTGGAGTGCT
	HvPRR1-3UTRv	GGGTTGAGGATGTATTGTGTGAGC
ELF3	HvELF3-Ex4Fw	AGGAAACCAGGCTTCACCATCTCA
	HvELF3UTRv	TTTCTTTCTTTCTGCCGGCCAACC
PPDH1	HvPpdH1-5UTRfw	TTCCAGGAGACGATTCATTCCGCT
	HvPpdH1-Ex4Fw	CAACAGCAATGATCGCAACGACGA
	HvPpdH1-Ex6fwB	TGCCATATCTTGAGCTGAGCCTGA
	HvPpdH1-Ex7Fw	TCCAGTGTTGTCAATCCTTCGGGT
	HvPpdH1-Ex1Rv	GGATGGTCTTCCTGTGTAGGAACT
	HvPpdH1-Ex6Rv	AAGTCAGCAGCTCGAACAATTGGC
	HvPpdH1-Ex7Rv	ACCCGAAGGATTGACAACACTGGA
	HvPpdH1-3UTRv	TATAGCTAGGTGCGTGGCGGGA
GI	HvGI-5UTRFw	TTCGCCTCTGCCTCAGCCTC
	HvGI-Ex7Fw	ACACCTCTGACCAGGCTACAACAA
	HvGI-Ex11Fw	ACTGCAATGTCTATGAGGGCTGGT
	HvGI-Ex12FwB	AACAGCACCAGCTCATCGGGTAAT
	HvGI-Ex4Rv	CGCTGTCAGATGTGAACTGACCAA
	HvGI-Ex7Rv	TTGTTGTAGCCTGGTCAGAGGTGT
	HvGI-Ex11Rv	AGACATTGCAGTTCCAATCGCACG
	HvGI-Ex12RvB	ATTACCCGATGAGCTGGTGCTGTT
	HvGI-Ex14Rv	ACAAGCATCCCATCTGTAGCACGA
	HvGI-3UTRv	AGACTGGGAATGGAAAGCCATCCT
PRR59	HvPRR59-Ex3Fw	CACGGTGCTAAAGTGCATGCAGAA
	HvPRR59-Ex5Fw	TAATGTGGCAAGCTCACCCAGAGA
	HvPRR59-Ex5RvB	TCTCTGGGTGAGCTTGCCACATTA
	HvPRR59-Ex6Rv	ACGTTGCTGCTTCCCTAAGTTGAC
	HvPRR59-Ex7RvB	CAAAGGAGCCTGAGCTTGCATTGT
PRR95	HvPRR95-5UTRFw	TGGTGGCTACTTGATCTGCGAGT
	HvPRR95-Ex4Fw	AAGAGAATGTCGCAGAACAGCACG
	HvPRR95-Ex4Rv	CGTGCTGTTCTGCGACATTCTCTT
	HvPRR95-Ex6Rv	GAGCCGCCCAATTTCCATTCTTGT
PRR73	HvPRR73-5UTRFw	AAACATCCACGGCTACTTTCACGC
	HvPRR73-Ex5Fw	AGTGTTCCAAACCAAAGACTGGTG
	HvPRR73-Ex7Fw	ACACAGAATCGGAACAGGCTGCTA
	HvPRR73-Ex8Fw	TCCAGCATCACCATCATGGACACT
	HvPRR73-Ex3Rv	CAAGGCATGGCGACCTCAGTTAAT
	HvPRR73-Ex7Rv	TAGCAGCCTGTTCCGATTCTGTGT
	HvPRR73-Ex8RvB	TCCATCTTGCAGGTGGAGTTCGTT
	HvPRR73-3UTRv	TGGCTACTGGCTTGCTCCTTCTTA
FKF1	HvFKF1-Ex2Fw	ATTCCTGCTTGACTTGACGAA
	HvFKF1-Ex2RvB	TAACTCTACATGGGACGTCG
