# Supplementary material for: Alternative Splicing of Barley Clock Genes in Response to Low Temperature
Source: PLoS One. 2016 Dec 13;11(12):e0168028. doi: 10.1371/journal.pone.0168028 (PMC5154542; doi:10.1371/journal.pone.0168028)
Supplement: S3 Table — (PDF) [file pone.0168028.s011.pdf]

Table S3. **AS events in barley clock and clock-related genes detected by HR RT-PCR experiments.**

Gene	Primer pair	Primer position	Product size (bp)	AS event name	Sequencing	AS event	Transcript abundance
LHY	HvLHY-Ex3Fw	5' UTR	278	FS	NGS, Sanger	FS	****
	HvLHY-Ex6Rv		268	Alt 3' ss E4	Sanger	Alt 3' ss E4 (-10 nt)	**
			294	Alt 3' ss I2	NGS, Sanger	Alt3' ss I2 (+16 nt)	*
			358	Alt 3' ss I1	NGS, Sanger	Alt 3' ss I1 (+80 nt)	**
			393	I2R	NGS, Sanger	Intron 2 retained	*
			440	I1R	Sanger	Intron 1 retained	-
			555	I1R + I2R	Sanger	Introns 1 and 2 retained	-
			666	I3R	Sanger	Intron 3 retained	-
			746	Alt 3' ss I1+ I3R	Predicted	Alt 3' ss I1 (+80 nt) and I3 retained	-
			781	I2R + I3R	Sanger	Introns 2 and 3 retained	-
			943	I1R, I2R + I3R	Sanger	Introns 1, 2 and 3 retained, unspliced	-
	HvLHY-Ex6Fw	MYB-coding	255	FS	NGS, Sanger	FS	****
	HvLHY-Ex8Rv	domain	235	Alt 5' ss E4	NGS	Alt5' ss E4 (-20 nt)	-
			325	I4R	NGS	Intron 4 retained	*
			490	I5R	Predicted	Intron 5 retained	-
			560	I4R + I5R	Predicted	Introns 4 and 5 retained, unspliced	-
	HvLHY-Ex8Fw	Span long intron 6	188	FS	NGS, Sanger	FS	****
	HvLHY-Ex9Rv		938	Alt E6a	NGS, Sanger	Alt E6a from intron 6 (+756 nt)	-
			1833	I6R	Unknown	Intron 6 retained	-
	HvLHY-Ex10FwB	C-terminus	916	FS	NGS	FS	****
	HvLHY-3UTRv	coding region	1005	I8R	NGS	Intron 8 retained, unspliced	**

Gene	Primer pair	Primer position	Product size (bp)	AS event name	Sequencing	AS event	Transcript abundance
PRR37	HvPpdH1-5UTRfw HvPpdH1-Ex1Rv	5’ UTR and coding region	250	FS	NGS, Sanger	FS	****
			194	Unknown	Unknown	Unknown	**
			230	Unknown	Unknown	Unknown	*
			246	Alt 3' ss E2	NGS	Alt 3' ss E2 (-4 nt)	-
			362	I1R	NGS, Sanger	Intron 1 retained	***
	HvPpdH1-Ex4Fw HvPpdH1-Ex6Rv	Exons 3 - 5	456	FS	NGS, Sanger	FS	***
			405	alt 5' and 3’ ss E6	NGS, Sanger	alt 5' ss E6 (- 45 nt) and alt 3' ss E6 (-6 nt)	***
			411	alt 5’ ss E6	Sanger	alt 5' ss E6 (- 45 nt)	***
			450	alt 3' ss E6	NGS, Sanger	alt 3' ss E6 (-6 nt)	***
			461	alt 5’ ss I6	NGS, Sanger	alt 5' ss I6 (+ 5 nt), adds PTC	***
			547	alt 3' ss E6 + I6R	Sanger	alt 3' ss E6 (-6 nt) and I6R	**
			553	I6R	Sanger	Intron 6 retained	*
	HvPpdH1-Ex6fwB HvPpdH1-3UTRv	C-terminus coding region	902	FS	NGS, Sanger	FS	****
			653	CrIn E8	Sanger	Cryptic Intron E8 (-249 nt), in frame	*
			984	I7R	NGS, Sanger	Intron 7 retained	**
PRR73	HvPRR73-5UTRFw HvPRR73-Ex3Rv	5’ UTR and N-terminus coding region	606	FS	NGS, Sanger	FS	****
			599	alt 3’ ss E2	Predicted	alt 3’ ss E2 (-7 nt), uORF decreased from 36 to 11 aa.	-
			861	alt 5’ ss I1 + alt E1a	NGS	alt 5’ ss Intron 1 and Alt E1a (+255 nt)	-
	HvPRR73-Ex3Fw HvPRR73-Ex5Rv	Exons 3 - 5	320	FS	NGS, Sanger	FS	****
			439	I4R	Predicted	Intron 4 retained	-
			465	I5R	NGS	Intron 5 retained	-
			584	I4R+I5R	Predicted	Introns 4 and 5 retained	-
	HvPRR73-Ex5Fw HvPRR73-Ex7Rv	Exons 5 and 7	549	FS	NGS, Sanger	FS	****
			632	I6R	NGS, Sanger	Intron 6 retained	*
	HvPRR73-Ex7Fw HvPRR73-Ex8RvB	Exons 7 and 8	319	FS	NGS, Sanger	FS	****
			404	I7R	NGS	Intron 7 retained	**

Gene	Primer pair	Primer position	Product size (bp)	AS event name	Sequencing	AS event	Transcript abundance
GI	HvGI-5UTRFw	5' UTR and	391	FS	NGS, Sanger	FS	****
	HvGI-Ex4Rv	N-terminus	251	E2S	NGS	E2 skipping	**
		coding region	263	E2S + Alt 3' ss I2	NGS, Sanger	E2 skipping and Alt 3' ss I2 (+12 nt)	***
			395	alt 5' ss E2 + alt 3' ss I2	NGS	alt 5' ss E2 (-8 nt) and alt 3' ss I2 (+12 nt)	***
			403	alt 3' ss I2	NGS	Alt 3' ss I2 (+12 nt)	***
			540	I2R	NGS, Sanger	Intron 2 retained	**
	HvGI-Ex12FwB	Exons 12 - 14	402	FS	NGS, Sanger	FS	****
	HvGI-Ex14Rv		549	I13R	NGS	Intron 13 retained	-
	HvGI-Ex14Fw	Exons 14 - 16	248	FS	NGS, Sanger	FS	****
	HvGI-Ex16Rv		336	I15R	NGS	Intron 15 retained	-
			407	I14R	NGS	Intron 14 retained	-
			495	I14R+I15R	Predicted	Introns 14 and 15 retained, unspliced	-
TOC1	HvPRR1-5UTRFwB	Exons 1 - 3	678	FS	NGS, Sanger	FS	****
	HvPRR1-Ex3Rv		840	I1R	Sanger	Intron 1 retained	-
	HvPRR1-Ex3Fw	Exons 3 - 6	463	FS	NGS, Sanger	FS	****
	HvPRR1-Ex6Rv		297	E4S	NGS, Sanger	E4 skipping	-
			593	I3R	NGS, Sanger	Intron 3 retained	*
			793	I3R+I4R	NGS	Introns 3 and 4 retained, unspliced	-
ELF3	HvELF3-Ex2Fw	Exons 2 - 4	338	FS	NGS, Sanger	FS	****
	HvELF3-Ex4Rv		468	I3R	NGS, Sanger	Intron 3 retained	*

Gene	Primer pair	Primer position	Product size (bp)	AS event name	Sequencing	AS event	Transcript abundance
CO2	HvCO2-Ex1FwB	C-terminus	509	FS	NGS, Sanger	FS	****
	HvCO2-3UTRv	coding region	579	Alt 3’ ss	Sanger	Alt 3’ ss (+68 nt, PTC)	***
			1004	CrIn	Sanger	Cryptic intron antisense strand	*
			1094	IR	NGS, Sanger	Intron retained, unspliced	***
PRR59	HvPRR59-Ex3Fw	Exons 3 - 5	309	FS	NGS, Sanger	FS	****
	HvPRR59-Ex5RvB		611	I4R	NGS	Intron 4 retained	-
			614	I3R	NGS	Intron 3 retained	-
	HvPRR59-Ex5Fw	Exons 5 - 7	673	FS	NGS, Sanger	FS	****
	HvPRR59-Ex7RvB		601	CrIn E6	NGS	Cryptic intron E6 (-72 nt, same ORF)	-
			866	I7R	NGS	Intron 7 retained	-
	HvPRR59-Ex7Fw	C-terminus	344	FS	NGS, Sanger	FS	****
	HvPRR59-3UTRv	coding region	470	I7R	NGS	Intron 7 retained	*
PRR95	HvPRR95-5UTRFw	N-terminus	610	FS	NGS, Sanger	FS	****
	HvPRR95-Ex4Rv	coding region	704	I2R	NGS	Intron 2 retained	-
	HvPRR95-Ex4Fw	Exons 4 - 6	612	FS	NGS, Sanger	FS	****
	HvPRR95-Ex6Rv		330	Unknown	Unknown	Unknown	-
			842	I4R	Predicted	Intron 4 retained	-
			932	I5R	Predicted	Intron 5 retained	-
			1171	I4R+I5R	Predicted	Introns 4 and 5 retained	-
	HvPRR95-Ex6FwB	C-terminus	363	FS	NGS, Sanger	FS	****
	HvPRR95-3UTRv	coding region	450	I6R	NGS	Intron 6 retained	-

Transcript abundance information is relative to WT plants grown under LD and 20 °C.
- Not visible or transcript level < 1% of total transcripts; * Transcript level between 1 and 2% of total transcripts; ** Transcript level between 2 and 10% of total transcripts; *** Transcript level between 10 and 50% of total transcripts; **** Transcript level equal or greater than 50% of total transcripts.
